# Genomic Epidemiology of *Campylobacter jejuni* Transmission in Israel

**DOI:** 10.3389/fmicb.2018.02432

**Published:** 2018-10-16

**Authors:** Assaf Rokney, Lea Valinsky, Jacob Moran-Gilad, Katleen Vranckx, Vered Agmon, Miriam Weinberger

**Affiliations:** ^1^Central Government Laboratories, Israel Ministry of Health, Jerusalem, Israel; ^2^Public Health Services, Israel Ministry of Health, Jerusalem, Israel; ^3^Department of Health Policy and Management, Faculty of Health Sciences, School of Public Health, Ben-Gurion University of the Negev, Be'er-Sheva, Israel; ^4^ESCMID Study Group for Genomic and Molecular Diagnostics, Basel, Switzerland; ^5^Applied Maths NV, Sint-Martens-Latem, Belgium; ^6^Infectious Diseases Unit, Assaf Harofeh Medical Center, Zerifin, Israel; ^7^Sackler Faculty of Medicine, Tel Aviv University, Tel Aviv, Israel

**Keywords:** whole genome sequencing, virulence factors/genetics, *Campylobacter jejuni*, phylogeny, humans, animals, one health

## Abstract

**Objectives:** *Campylobacter jejuni* is responsible for 80% of *Campylobacter* infections in Israel, a country with a high incidence reaching 91/100,000 population. We studied the phylogeny, diversity and prevalence of virulence factors using whole genome sequencing (WGS) of a national sample of *C. jejuni* clinical, food, and animal isolates collected over a 10-year period (2003–2012).

**Methods:** *C. jejuni* isolates (*n* = 263) were subject to WGS using Illumina sequencing (PE 250bpx2). Raw reads and *de novo* assemblies were analyzed with the BioNumerics whole genome MLST (wgMLST) pipeline. Reads were screened for 71 virulence genes by the SRST2 script. Allelic profiles were analyzed to create minimum spanning trees and allelic core distances were investigated to determine a reliable cutoff for strain determination.

**Results:** wgMLST analysis of 263 *C. jejuni* isolates indicated significant diversity among the prevalent clonal complexes (CCs) with CC-21 and CC-353 being the most diverse, and CC-574 the most clonal. Within CC-21, sequence type (ST)-1359 created a separate clade. Human, poultry and bovine isolates clustered together across the different STs. Forty four percent of studied isolates were assigned to 29 genetic clusters. Temporal and geographical relatedness were found among the minority of clusters, while most phylogenetically associated cases appeared diffuse and unassociated epidemiologically. The majority of virulence factors were highly prevalent across the dataset and not associated with genotype, source of isolation or invasiveness. Conversely, all 13 genes associated with type VI secretion system (T6SS) were lineage-related and identified in only 18% of the isolates. T6SS was detected in 95.2% of ST-1359, a common type in Israel.

**Conclusions:** wgMLST supported the assessment that poultry and cattle are likely food sources of infection in Israel. Substantial genetic clustering among *C. jejuni* isolates suggested multiple point source and diffuse outbreaks that were previously unreported in Israel. The high prevalence of T6SS among ST-1359 isolates is unique to Israel, and requires further investigation. This study exemplifies the importance of studying foodborne pathogens using advanced genomic approaches across the entire spectrum of One Health.

## Introduction

*Campylobacter* species are major causes of foodborne infections globally and cause considerable morbidity, with the heaviest burden on young children (Weinberger et al., 2013; Kaakoush et al., 2015). In a recent analysis by the World Health Organization (WHO)-established Foodborne Disease Burden Epidemiology Reference Group (FERG), *Campylobacter* spp. were rated second (after Norovirus) among the causes of foodborne infections, responsible for 96 million cases globally (Kirk et al., 2015).

In Israel *Campylobacter* infections pose a major challenge to public health due to a high incidence reaching 91/100,000 population overall and up to 363/100,000 in children <1 year old. Similar to many other settings, *Campylobacter jejuni* is responsible for ~80% of these illnesses in Israel (Weinberger et al., 2013). Recently, multilocus sequence typing (MLST)-based phylogeny of a national sample of *C. jejuni* isolates in Israel, showed high diversity with a high proportion of novel sequence types (STs) (Weinberger et al., 2016). Although the food sources of *C. jejuni* infections in Israel were not fully investigated, poultry handling and consumption is considered a major source (Weinberger et al., 2013; Bassal et al., 2016). Indeed, in the above MLST-based analysis (Weinberger et al., 2016) most human, poultry and bovine STs clustered together in the leading clonal complexes (CCs), suggesting that poultry and cattle were likely food sources of clinical infection in Israel.

While MLST has been an essential research tool for studying the epidemiology of *C. jejuni* infections and has greatly contributed to current knowledge on human infections and potential sources, it is less suitable for routine, real-time public health surveillance (Deng et al., 2016; Llarena et al., 2017; Moran-Gilad, 2017). Whole genome sequencing (WGS) is currently considered a much more powerful method to investigate foodborne infections and outbreak*s* since it provides a high power of discrimination and high epidemiologic concordance (Nadon et al., 2017). Indeed, WGS has been successfully used on multiple occasions in the investigation of local and international foodborne outbreaks due to *Listeria monocytogenes* (Jackson et al., 2016; Kwong et al., 2016; Chen et al., 2017; Schjorring et al., 2017), Shiga toxin-producing *Escherichia coli* (Butcher et al., 2016; Moran-Gilad et al., 2017; Mylius et al., 2018), as well as *Campylobacter* spp. (Revez et al., 2014; Fernandes et al., 2015; Lahti et al., 2017). WGS was also applied to the study of nosocomial outbreaks due to resistant bacteria, most notably the investigation of carbapenem-resistant *Klebsiella pneumoniae* spread in hospitals as well as its role in endoscope-associated outbreaks (Marsh et al., 2015; Zhou et al., 2017). Moreover, the cumulative experience with the performance of WGS in the US for the investigation of foodborne outbreaks and in pinpointing sources and reservoirs led to the switch from Pulsed-Field Gel Electrophoresis (PFGE) foodborne surveillance network to WGS-based network—GenomeTrakr for real-time surveillance (Allard et al., 2018). Additionally, the transition from traditional to WGS-based workflow in microbiology laboratories allows to retrieve, in a single step, information about the genotype and the subtype of the foodborne pathogen, its resistance genes and the profile of the virulence factors (Allard et al., 2018).

Applying WGS to the routine surveillance of *C. jejuni* is somewhat more challenging than other foodborne organisms. *C. jejuni* is a genetically unstable pathogen with high inter and intra-species recombination rates that lead to a weakly clonal population. High levels of diversity have been reported in several genomic studies (Llarena et al., 2017). Notably, more studies are needed to establish epidemic cutoffs for identifying and validating links between human, veterinary and environmental isolates.

Profiling the virulence factors in *C. jejuni* may be also helpful to gain a better understanding of the pathogenicity of the microorganism and possibly aid in implementing control measures, especially if factors such as heat/cold stability or lability are discovered (Allard et al., 2018).

The type VI secretion system (T6SS) is an effector translocation apparatus identified in a variety of Gram-negative bacteria. This system has been implicated in a broad range of bacterial functions including virulence, metabolism and antimicrobial resistance and contributes to adaptation and host interactions. This system consists of 13 loci (TagH, TssA–TssG, TssI-TssM) shown to confer competitive advantage and contribute to environmental adaptation in several species. A functional T6SS has been discovered in *C. jejuni, C. coli*, and was shown to confer contact-dependent cytotoxicity toward red blood cells (Lertpiriyapong et al., 2012; Bleumink-Pluym et al., 2013). Several reports identified T6SS in the *C. jejuni* and *C. coli* populations (Corcionivoschi et al., 2015; Siddiqui et al., 2015; Ugarte-Ruiz et al., 2015), though linkage to genotypes has not been studied in detail. A recent report has identified this system on a plasmid in *C. coli* suggestive of possible mobility within *Campylobcater* and transmission to other species (Ghatak et al., 2017).

Here we report a WGS-based analysis of a national sample of *C. jejuni* clinical and veterinary isolates with focus on the genomic epidemiology and the virulence characteristics of this microorganism in Israel.

## Materials and methods

Two hundred and sixty three *C. jejuni* isolates representing the major lineages in Israel as identified by MLST (Weinberger et al., 2016) and collected over a 10 year period (2003–2012) were analyzed by WGS (Supplementary Table 1). DNA extraction was performed on the automated QIAsymphony SP platform (Qiagen, Hilden, Germany), and quantified by the Qubit 2.0 Fluorometer (Life Technologies, Carlsbad, CA, USA). DNA libraries were prepared from 1 ng of purified DNA using Nextera XT DNA Sample Prep Kit and Nextera XT Index Kit (Illumina, Inc., San Diego, CA, USA). Libraries were subject to short read sequencing PE 250bpx2, aiming at >100X coverage. *De novo* assembly and whole genome MLST (wgMLST) analysis were performed on the BioNumerics 7.6 (Applied Maths, Belgium) calculation engine using default settings. The reads were *de novo* assembled by SPAdes 3.7.1, annotated, and wgMLST analysis of assembly-free, and assembly-based allele calls was performed (Bankevich et al., 2012). Quality metrics of reads and assemblies are detailed in Supplementary Table 1. Phylogeny was inferred by calculating a minimal spanning tree based on wgMLST or MLST allelic profiles. Diversity within CCs was estimated by the distribution of allelic differences in the wgMLST distance matrix. Identification of potential clusters of human isolates from major STs was based on a cutoff of ≤15 wgMLST allele difference (Schürch et al., 2018).

Virulence factors reported in the literature (Supplementary Table 2) were extracted from reference strain NCTC11168 (AL111168.1). The 13 genes that constitute the type VI secretion system (T6SS) in *C. jejuni* were extracted from strain 108 (JX436460.1). Reads were screened for the presence of virulence genes by the SRST2 script with default parameters (Inouye et al., 2014). The presence of genes was defined as DNA identity and coverage of ≥90%.

The pubMLST *C. jejuni* database was screened for the hemolysin co-regulated protein (*hcp)* gene, the hallmark of T6SS. All 10,906 deposited *C. jejuni* sequences >1 Mb (as of Feb 18th, 2018) were searched for the presence of the *hcp* sequence by BLASTN (NCBI, NIH) of the DNA sequence from *C. jejuni* strain 108. Presence of the gene was determined by >90% alignment and identity with the query sequence (*e*-value = 0). A BLASTN search of the *hcp* sequence was similarly performed on *de novo* assemblies of Israeli isolates.

### Ethical consideration

The study was approved by the local ethical committee at Assaf Harofeh Medical Center, Zerifin, Israel and the local ethical committee of Tel Aviv University, Tel Aviv, Israel.

## Results

**Figure 1:**
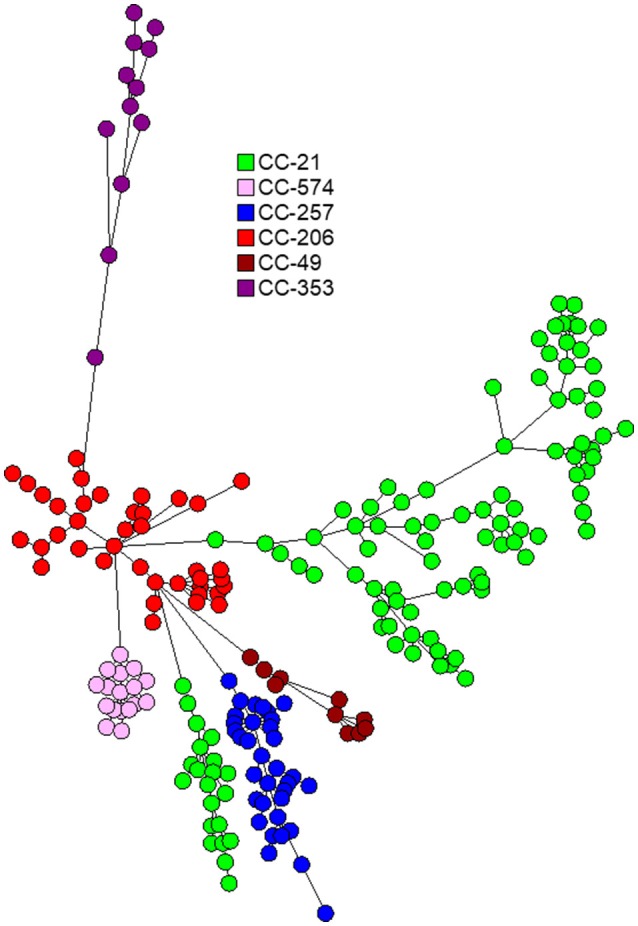
Phylogenetic analysis of *C. jejuni* isolates from the six leading clonal complexes in Israel during 2003–2012. The minimum spanning trees are based on wgMLST analyses of 192 clinical and 24 veterinary isolates. Isolates are represented by circles connected by branches proportional to the allelic distance. Colors represent clonal complexes.

**Figure 2:**
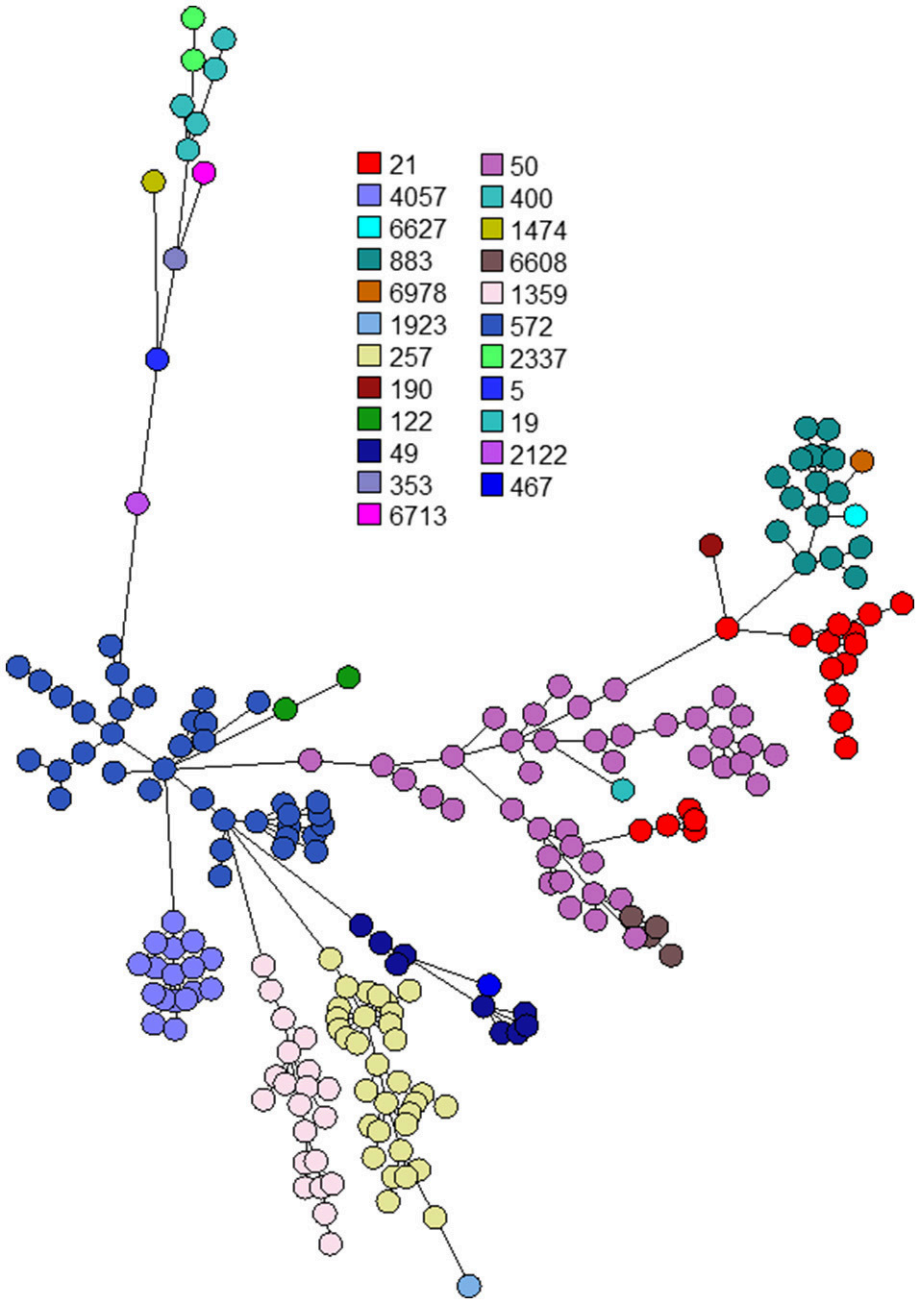
Phylogenetic analysis of *C. jejuni* isolates from the six leading clonal complexes in Israel during 2003–2012. The minimum spanning trees are based on wgMLST analyses of 192 clinical and 24 veterinary isolates. Isolates are represented by circles connected by branches proportional to the allelic distance. Colors represent sequence types.

**Figure 3:**
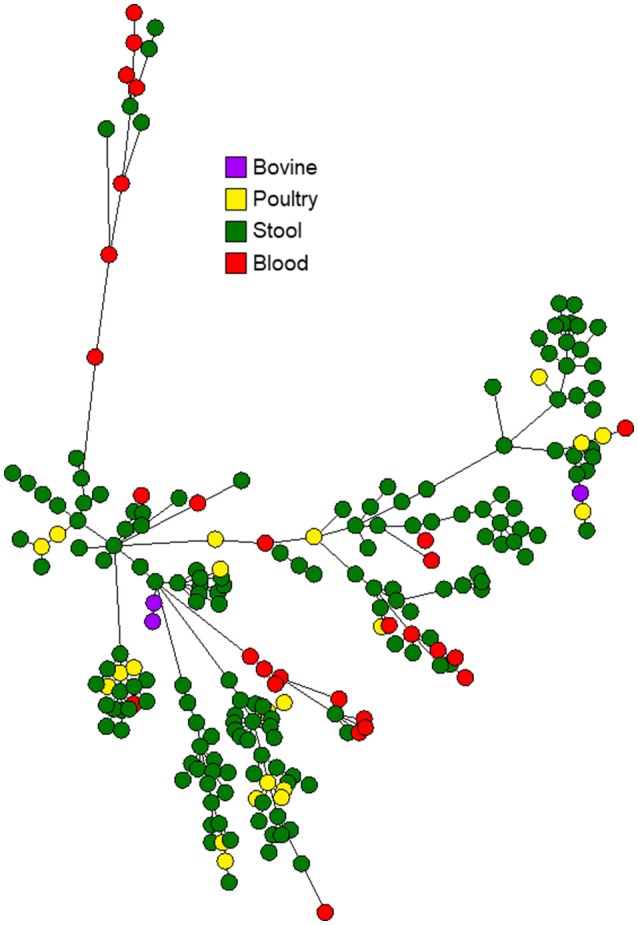
Phylogenetic analysis of *C. jejuni* isolates from the six leading clonal complexes in Israel during 2003–2012. The minimum spanning trees are based on wgMLST analyses of 192 clinical and 24 veterinary isolates. Isolates are represented by circles connected by branches proportional to the allelic distance. Colors represent isolation source.

**Figure 4:**
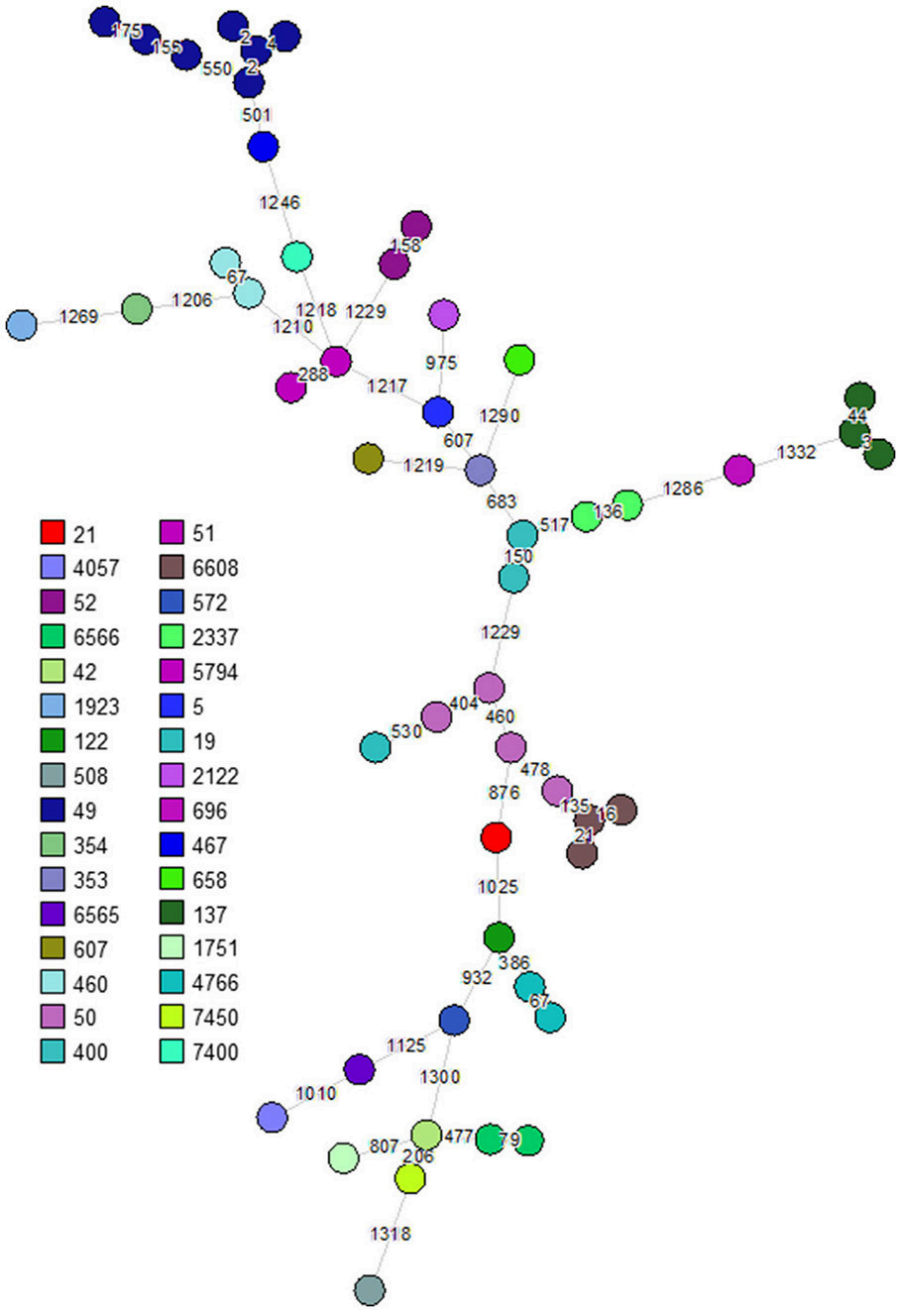
Phylogenetic analysis of *C. jejuni* isolates from 51 bacteremic cases. A minimum spanning tree based on wgMLST analysis of isolates from blood source. Isolates are represented by circles connected by branches proportional to the allelic distance. Colors represent sequence types, branches and numbers represent allelic differences between isolates.

The 263 *C. jejuni* studied isolates were assigned to 51 STs and 18 CCs (Supplementary Table 1). The dataset included 239 human isolates (188 from stool, 51 from blood), 21 poultry isolates and 3 bovine isolates. Six CCs (CC-21, CC-206, CC-257, CC-574, CC-353, CC-49) were responsible for 82.1% of the isolates. The wgMLST analysis indicated significant diversity among the top six CCs (Figure 1). CC-21 appeared most diverse, particularly with respect to its members ST-21 and ST-50 (Figures 2). Across the different STs, human stool and blood isolates, as well as poultry and bovine isolates clustered together (Figure 3). Blood isolates were assigned to 32 STs, grouped into 16 CCs (Figure 4). A few blood isolates within ST-137 and ST-49 were as close as 2–4 alleles apart, while all other isolates were more widely dispersed.

**Figure 5:**
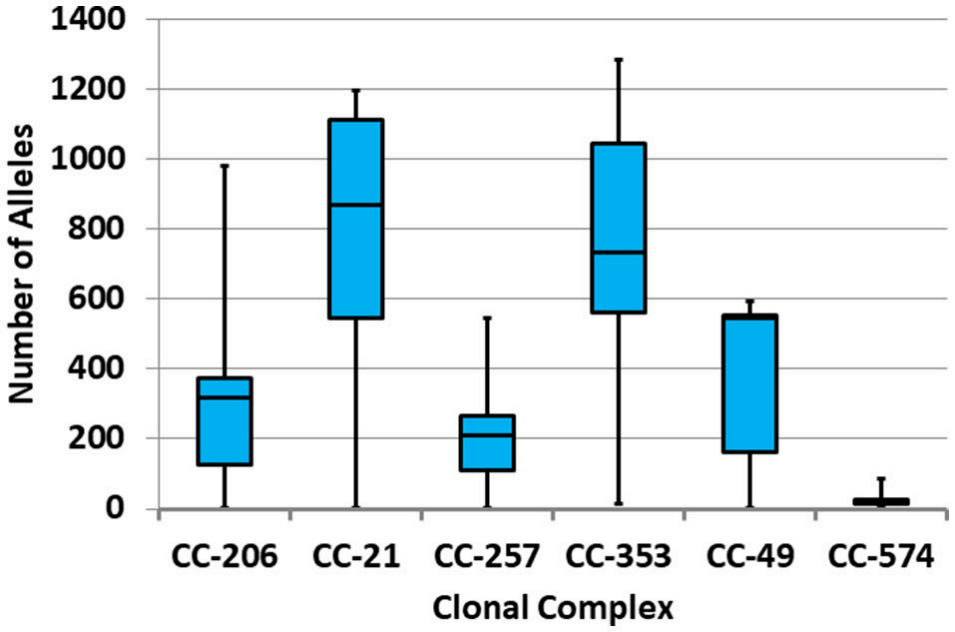
Distribution of allelic distances within the six leading clonal complexes. The distribution of allelic differences between each pair of isolates is presented in a box-whisker plot.

Diversity within CCs and STs was further analyzed by the range and distribution of allelic differences (Figure 5). CC-21 and CC-353 were the most diverse CCs, while CC-574 was the most clonal. Within CC-21, ST-1359 formed a separate clade consisting of human stool and poultry isolates. ST-1359 was clonal and distant (≥1,071 alleles) from the rest of the CC-21 isolates.

For each veterinary isolate the closest human isolate was identified by calculating the allelic distances (Table 1). Eight of 21 poultry isolates (38.1%) and one of three bovine isolates (33.3%) had a genetically close human isolate (defined as having no more than 15 alleles apart). For most of the other veterinary isolates, the closest human isolate was 25–56 alleles apart. Three poultry isolates had allelic distances of 121, 286, and 353, respectively, to the closest human isolates. Allelic distance between veterinary and human isolates varied across the same ST.

**Table 1:** Genetic relatedness between veterinary and closest human *C. jejuni* isolates.

**Veterinary source**	**Isolate**	**ST**	**CC**	**Closest human strain, isolate no**.	**Allelic distance**
Poultry	169p	257	CC-257	56971	3
	191p	257	CC-257	56971	3
	208p	21	CC-21	60663	5
	68a	50	CC-21	90249	5
	9lul	4057	CC-574	88796	8
	95p	4057	CC-574	47845, 67665	9
	119p	4057	CC-574	67665	10
	211p	1359	CC-21	53818	13
	36	572	CC-206	93196	25
	40	572	CC-206	93196	30
	56	572	CC-206	60230	37
	6	257	CC-257	75914	38
	5	257	CC-257	75914	39
	140	257	CC-257	75914	39
	210p	1359	CC-21	67219	44
	189	21	CC-21	91626	52
	28	257	CC-257	75914	55
	29	21	CC-21	91626	56
	151	883	CC-21	76564	121
	31b	50	CC-21	44845	286
	94	50	CC-21	73257	353
Bovine	9c	21	CC-21	56786, 60663	9
	78c	572	CC-206	35992	45
	45c	572	CC-206	35992	42

Human isolates were also analyzed for allelic distances and for genetic clusters within the same ST. A total of 104 out of 239 (43.5%) human isolates were assigned into 29 different clusters (Table 2). Most clusters were spread over several study years, and one cluster (ST-4057 cluster Y) was spread over the whole study period. None of the isolates within a cluster shared the same neighborhood, as they were characteristically spread all over the country. Within a cluster, there was no association between the range of allelic distances and the year range. Clusters formed within <30 days were further investigated. Eleven pairs of isolates were identified, some within larger clusters (Table 3). The allelic distances between the pairs ranged from 1 to 10 (mean 4.8). In two clusters the patients were geographically linked (same city, but not the same neighborhood), in another two—they resided <50 kilometers apart, and in the rest—>50 kilometers apart. In most clusters the patients' ages also diverged.

**Table 2:** Characterization of genetically related clusters among human *C. jejuni* isolates.

**Sequence type (No. isolates)**	**Cluster**	**No. isolates in a cluster**	**Allelic distance**	**Timeframe**
ST-50 (38)	A	2	2	2008, <30 d
	B	2	7	2004–2005, <1 y
	C	2	12	2004–2012
	D	3	10	2003–2005
	E	2	9	2009–2012
	F	2	4	2009 <30 d
	G	2	6	2005–2006
	H	2	7	2008, >30 d
	I	3	8	2006–2008
ST-572 (32)	J	2	1	2007–2008
	K	5	6–12	2003–2009
	L	4	1–6	2011–2012
	M	3	2–3	2010–2011
ST-257 (27)	N	4	3–17	2008–2010
	O	6	3–15	2004–2007
	P	2	7	2010–2012
	Q	10	1–10	2007–2012
ST-1359 (19)	R	5	2–9	2006, 0–>30 d
	S	2	8	2006–2008
	T	2	8	2007, >30 d
ST-883 (16)	U	2	5	2010–2011, <30 d
	V	5	7–14	2004–2009
ST-21 (14)	W	8	5–12	2006–2010
	X	5	7–10	2006–2008
ST-4057 (14)	Y	9	4–13	2003–2012
	Z	2	13	2010, >30 d
ST-49 (9)	AA	4	2–4	2006–2012
ST-400 (5)	AB	2	13	2006–2010
ST-460 (5)	AC	2	9	2006, <30 d

*d, days; y, years*.

**Table 3:** Analysis of genetically related *C. jejuni* isolates clustered in time (allelic distance ≤ 15, temporal relatedness ≤ 30 days).

**Sequence type**	**Cluster**	**Allelic distance**	**Dates of isolation**	**Geographic distance**	**Age band**	Ethnicity^*^
ST-50	A	2	13/01/2008, 13/01/2008	>50 Km	0– < 10, 70– < 80	Same
	F	4	16/08/2009, 25/08/2009	>50 Km	10– < 20, 0– < 10	Same
	I	8	21/05/2006, 07/06/2006	>50 Km	0– < 10, 20– < 30	Same
ST-572	L	1	17/07/2012, 24/07/2012	Same city	0– < 10, 0– < 10	Same
	M	5	15/03/2011, 28/03/2011	Same city	70– < 80, 20– < 30	Same
ST-257	N	3	30/11/2010, 13/12/2010	>50 Km	0– < 10, 0– < 10	Different
	O	3	22/05/2006, 30/05/2006	>50 Km	50– < 60, 10– < 20	Same
	Q	10	12/11/2007, 10/12/2007	>50 Km	60– < 70, 10– < 20	Same
ST-1359	S	2	13/12/2006, 13/12/2006	< 50 Km	0– < 10, 0– < 10	Different
ST-21	X	9	04/06/2006, 28/06/2006	< 50 Km	0– < 10, 60– < 70	Different
ST-460	AC	9	31/01/2006, 23/02/2006	>50 Km	0– < 10, 0– < 10	Same

* *Jewish or non-Jewish ethnicity*.

**Figure 6:**
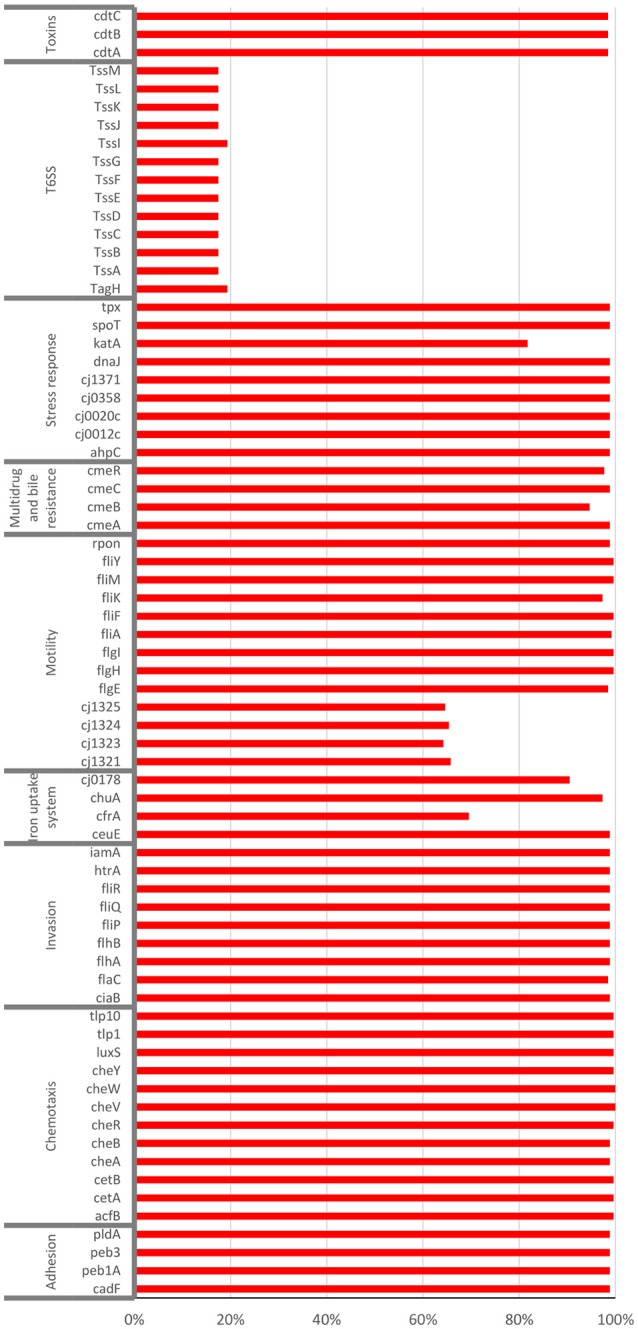
Prevalence of recognized virulence factors in 263 *C. jejuni* isolates. Illumina reads were screened for the presence of 71 virulence and survival factors by the SRST2 script. The presence of each factor was determined by >90% identity with the query sequence from strain NCTC11168.

The sequences of 263 *C. jejuni* isolates were screened for 71 genes recognized as virulence and environmental adaptation factors (motility, chemotaxis, T6SS, stress response, invasion, toxins, iron uptake, adhesion, and multidrug and bile resistance). The majority of the virulence genes were highly prevalent in the dataset and were not linked to a specific ST or CC (Supplementary Figure 1 and Figure 6). The virulence factors were similarly distributed among human and veterinary isolates as well as among isolates of blood or stool source (Supplementary Figure 1).

**Figure 7:**
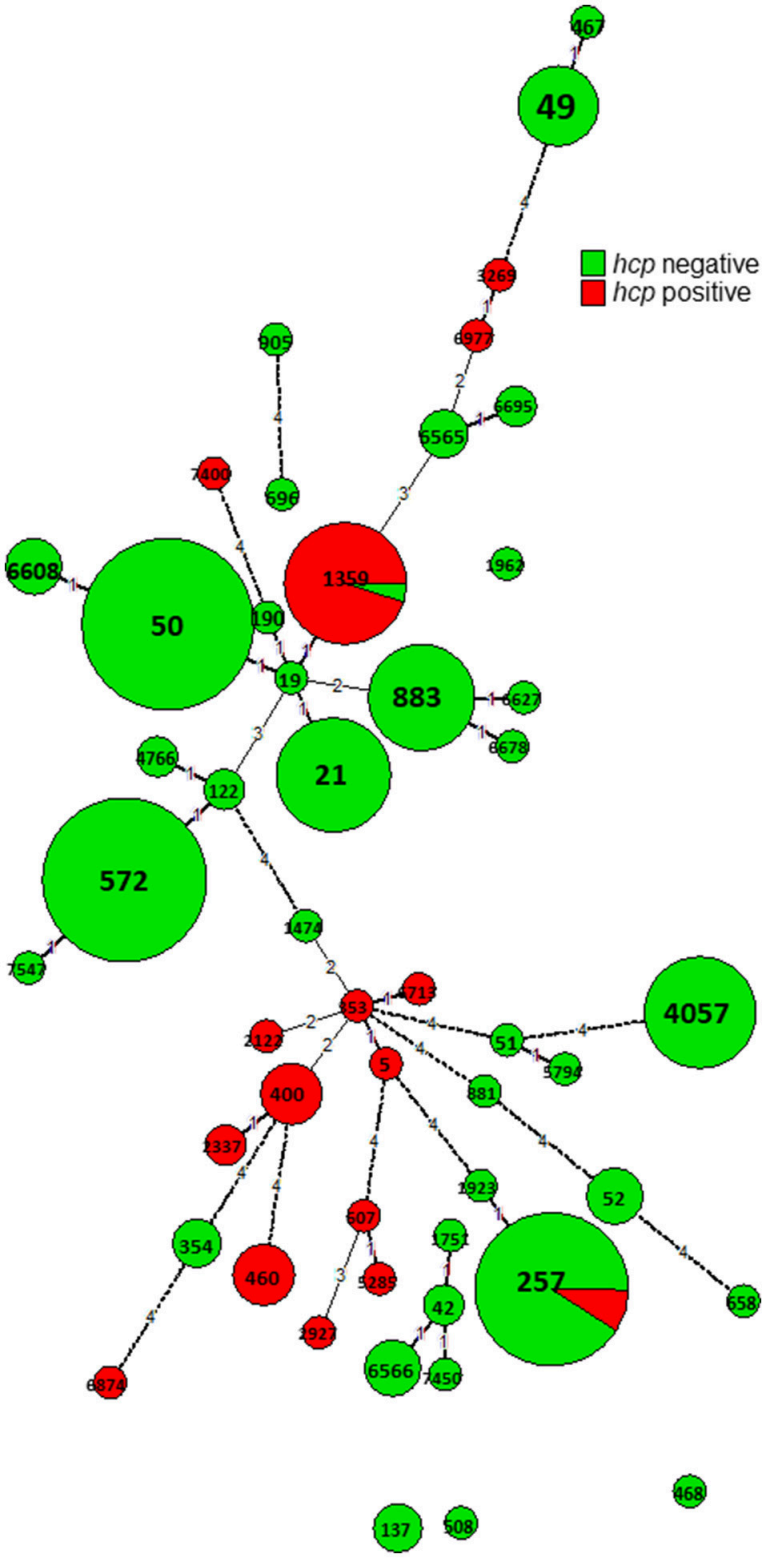
Presence of hemolysin co-regulated protein (*hcp*) gene among Israeli *C. jejuni* isolates. A minimum spanning tree based on wgMLST was extracted from the WGS. The sequences were screened for the presence of the *hcp* (TssD) sequence by BLASTN.

In contrast, all 13 genes associated with T6SS were lineage related and identified in only 17% of the isolates. A high prevalence of genes associated with T6SS, reaching >90%, was found in the following CCs: CC-353, CC-460, CC-607, and CC-446 (Supplementary Figure 1 and Figure 7). The presence of the hcp marker (TssD) fully predicted the presence of all 13 genes associated with this system, as previously reported (Corcionivoschi et al., 2015; Ugarte-Ruiz et al., 2015).

**Figure 8:**
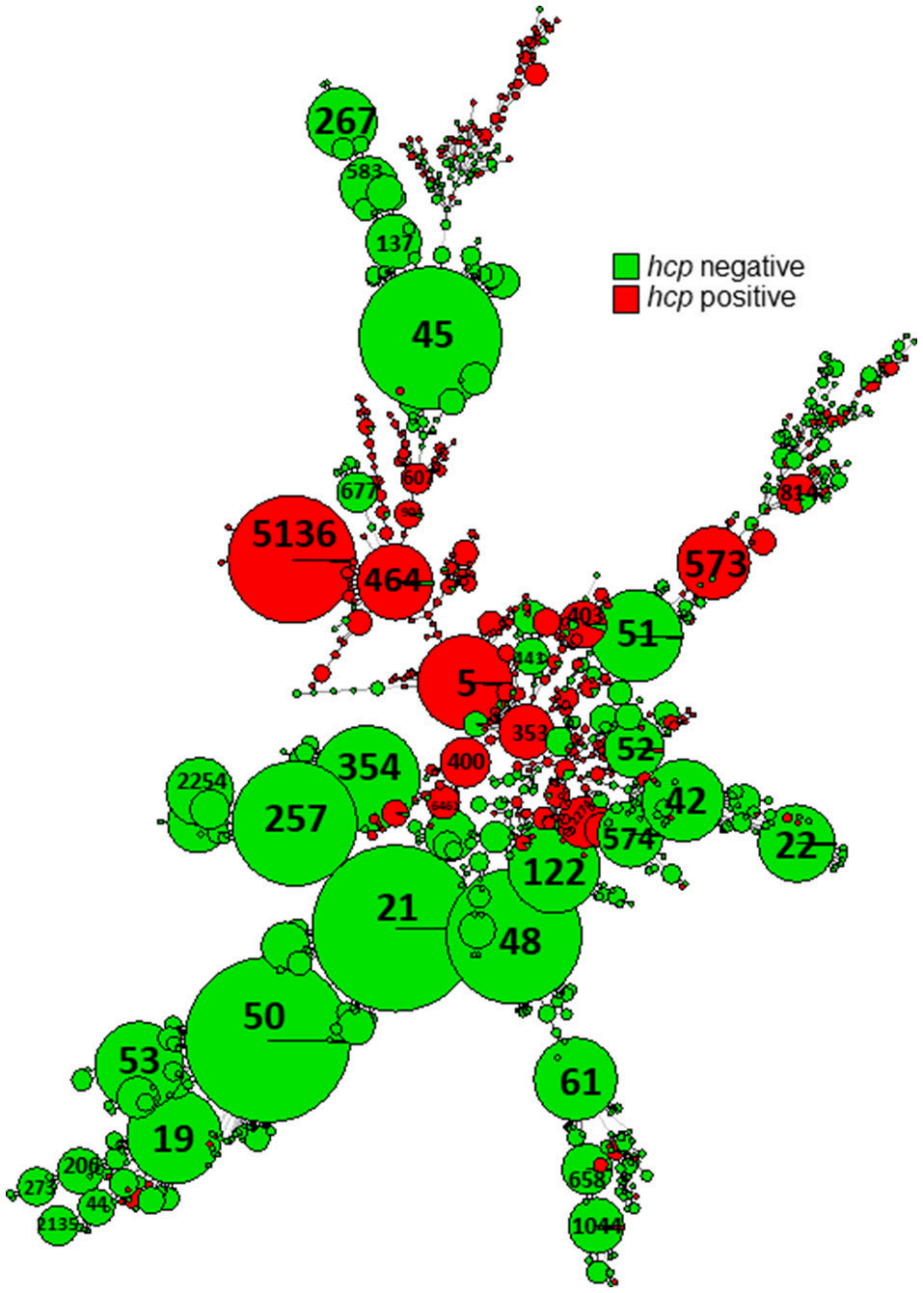
Presence of hemolysin co-regulated protein (*hcp*) gene in pubMLST sequences of global *C. jejuni* isolates. A minimum spanning tree based on wgMLST analysis of isolates submitted to pubMLST with WGS sequences available. The sequences were screened for the presence of the *hcp* (TssD) sequence by BLASTN.

Screening all genomic sequences of *C. jejuni* with an assigned ST available in pubMLST for the *hcp* gene (a valid predictive marker of T6SS) revealed its presence in 22% of 10,906 sequences (Table 4 and Figure 8). There was a high degree of congruence regarding the prevalence of *hcp* genes across STs between the global sample and the studied Israeli sample (Table 4). The only incongruence concerned CC-21, where Israeli isolates revealed substantially higher prevalence (19%) of *hcp* genes compared to the global collection of isolates (0.9%), and this was due to ST-1359 with 95.2% prevalence (Supplementary Figure 1).

**Table 4:** The prevalence of hemolysin co-regulated protein (*hcp)* gene among global and Israeli clonal complexes of *C. jejuni*.

**Clonal complex**	**Global sequences**		**Israeli sequences**	
	**No. isolates studied**	**Positive for *hcp* gene (%)**	**No. isolates studied**	**Positive for *hcp* gene (%)**
CC-446	19	100	2	100
CC-1275	12	100	–	–
CC-464	691	99	–	–
CC-607	82	99	3	100
CC-573	187	97	–	–
CC-460	63	95	5	100
CC-403	127	94	–	–
CC-353	697	89	12	92
CC-952	8	75	–	–
CC-661	80	55	–	–
Unassigned	775	49	13	15
CC-692	33	45	–	–
CC-702	9	44	–	–
CC-179	14	36	–	–
CC-828	90	23	–	–
CC-52	160	19	4	0
CC-1034	57	18	–	–
CC-433	8	13	–	–
CC-574	170	11	17	0
CC-1332	14	7	1	0
CC-354	405	6	3	0
CC-658	203	5	1	0
CC-257	785	3	34	9
CC-443	291	1	2	0
CC-21	2671	1	104	19
CC-22	183	1	–	–
CC-206	538	0	39	0
CC-48	660	0	1	0
CC-45	1023	0	3	0
CC-42	266	0	8	0
CC-61	236	0	–	–
CC-283	156	0	–	–
CC-677	59	0	–	–
CC-49	55	0	10	0
CC-508	53	0	1	0
CC-362	12	0	–	–
CC-177	6	0	–	–
CC-1287	5	0	–	–
CC-682	2	0	–	–
CC-1347	1	0	–	–
Total	10,906	22	263	17

## Discussion

This study analyzed the genomic epidemiology and virulence of *C. jejuni* in Israel using WGS. For this purpose, we studied human and veterinary isolates derived from a national sample that was previously typed by MLST. wgMLST-based phylogeny revealed a notable diversity within some CCs, particularly CC-21 and CC-353. Other CCs, such as CC-574 were more clonal. Allelic differences within a CC ranged from <10 to several hundreds of alleles, with occasional distances within a CC being greater than the distances between CCs. A similar study from Denmark (Kovanen et al., 2014) analyzed diversity within STs, and likewise found highly diverse and minimally diverse STs. The most diverse (>400 alleles apart) ST in that study was ST-45 within CC-45. CC-21 was not detected in that study. Notably, ST-45 was not found among the Israeli isolates, although it is commonly isolated in the UK and Europe (Colles and Maiden, 2012; Weinberger et al., 2016). The relative frequency of *C. jejuni* genotypes as well as their diversity may differ between countries and are probably influenced by multiple factors including the food sources, animal reservoirs, the timing of introduction into the country, the timeline and the rates of recombination and zoonotic transmissions (Dearlove et al., 2016).

Despite the high burden of *Campylobacter* infection in many countries, there is a dearth of information regarding relatedness of isolates using the highly discriminatory WGS-based subtyping (Joensen et al., 2018). Using a threshold of 15 alleles, we found that 43.5% of the studied isolates belonged to genetically-related clusters. However, the majority of these clusters were temporally spread over several years, and spatially spread countrywide. When zooming on clusters of isolates that were also temporarily related (within < 30 days), only 4 out of 11 were also spatially related. This finding may suggest that some outbreaks are probably derived from common, centrally distributed sources that are consumed throughout the country. Genetically-related clones, perhaps the more successfully adapted clones, may be circulating over several years. Nevertheless, similarity thresholds for this microorganism should be further studied and analyses should always consider epidemiological data in conjunction with WGS results.

Our study was based on a representative sample of <50 isolates per annum out of 6,000 to 8,000 yearly laboratory-documented human infections over a 10-year period. This sampling ratio appeared sufficient for detection of genetically related isolates, suggesting that large scale outbreaks may occur more often than previously appreciated.

In a similar analysis from Denmark, 25% of 245 studies isolates over 9 months, formed genetic clusters, mostly belonging to temporal and/or geographical clusters. Temporal relatedness in this study was defined as a 12-day interval. Some of the isolates were derived from epidemiologically defined suspected foodborne outbreaks, and the WGS confirmed their genetic relatedness. In that study genetic relatedness was determined by single nucleotide polymorphism (SNP) comparisons, and the cutoff was 5 SNPs. The current study from Israel and the Danish one, represent two major well-acknowledged strategies for defining genomic relatedness, being a gene-by-gene and a SNP-based approach (Schürch et al., 2018). wgMLST, used herein is advantageous for routine surveillance owing to its portability and scalability and strain nomenclature, which facilitate global data sharing for public health purposes (Nadon et al., 2017).

Several multifactorial systems in *Campylobacter* contribute to pathogenicity and virulence in humans, and to survival in broilers and throughout the food chain (Bolton, 2015). Our finding that multiple virulence factors are highly prevalent in *C. jejuni* is with agreement with previous studies (Koolman et al., 2015; Cantero et al., 2017). The exception is T6SS that is lineage-related. Previous reports have identified T6SS in subsets of isolates (Corcionivoschi et al., 2015; Siddiqui et al., 2015; Ugarte-Ruiz et al., 2015) but lineage relatedness has not been studied in detail. We found that similar to global *C. jejuni*, T6SS was restricted to selected Israeli STs, comprising 17% of all studied isolates. There was also an agreement regarding the CCs with the highest prevalence, being 353, 460, 607, and 446 in Israel. The exception that stood out was the high prevalence of T6SS in ST-1359 isolates (95.2%) in Israel, but not among the other isolates belonging to CC-21. Overall, T6SS was present in 0.9% of the global CC-21 isolates submitted to pubMLST and in 19% of the studied Israeli CC-21 isolates, due to the high prevalence in ST-1359. Notably, while ST1359 is prominent in Israel, only 11 isolates of ST-1359 are reported in pubMLST (0.11% of submitted *C. jejuni* isolates). Of them, two isolates isolated from the stool of patients from UK had sequences that could be analyzed, and both contained T6SS (IDs 59632, 69325).

Another important finding in our study was the genetic relatedness of the poultry and bovine isolates to human isolates. Overall, 38.1% of the 21 studied poultry isolates had a human isolate at a distance of <15 alleles, and 85.7% at a distance of <100 alleles. Similarly, one of the three studied bovine isolates had a human isolate at a distance of <15 alleles, and the other two at a distance of <100 alleles. This finding and the uniform pattern of virulence factor prevalence across STs are a strong argument that poultry and cattle are probable food sources of human *C. jejuni* infections in Israel. Unlike the situation in Finland (Feodoroff et al., 2013), we could not detect any dominant clone among the blood isolates despite the higher discriminative power of WGS. Furthermore, blood isolates did not differ from stool isolates concerning the prevalence of the screened virulence factors. This suggests that genetic factors, other than simply presence or absence of virulence gene may determine strain invasiveness.

In conclusion, whole genome analysis further supported the assessment that poultry and cattle are likely food sources of *C. jejuni* infection in Israel with isolates being able to persist over years throughout the food chain. The Israeli isolates harbored various virulence-associated genes, though the pattern could not be linked to properties of the isolates (invasiveness). Only the T6SS was linked to specific lineages. This study exemplifies the importance of studying foodborne pathogens using advanced genomic approaches across the entire spectrum of One Health and its results are expected to facilitate public health interventions across multiple sectors in order to mitigate the significant increase of campylobacteriosis in Israel over the last decade.

## Author contributions

AR, LV, JM-G, VA, and MW conceived the study. AR and LV carried out the wet lab work. AR, LV, KV, and MW performed the analyses. AR and MW drafted the manuscript with help from the other authors. JM-G critically revised the data and the manuscript. All authors approved of the final manuscript.

### Conflict of interest statement

The authors declare that the research was conducted in the absence of any commercial or financial relationships that could be construed as a potential conflict of interest.
