# Supplementary material for: Genomic Epidemiology of Campylobacter jejuni Transmission in Israel
Source: Front Microbiol. 2018 Oct 16;9:2432. doi: 10.3389/fmicb.2018.02432 (PMC6198274; doi:10.3389/fmicb.2018.02432)
Supplement: Supplementary file 1 [file Table_1.DOCX]

**SUPPLEMENTARY TABLE 1. *C. JEJUNI* VIRULENCE AND SURVIVAL FACTORS INCLUDED IN THIS STUDY**

| **Group** | **Encoding gene** | **Virulence factor** | **Accession** | **Start** | **End** | **Locus Tag** |
| --- | --- | --- | --- | --- | --- | --- |
| Adhesion | cadF | Outer membrane protein | AL11168.1 | 1413913 | 1414872 | Cj1478c |
|  | peb1A | Peb1, periplasmic binding protein | AL11168.1 | 857098 | 857877 | Cj0921c |
|  | peb3 | Peb3, transport protein | AL11168.1 | 266622 | 267374 | Cj0289c |
|  | pldA | Phospholipase A | AL11168.1 | 1282933 | 1283922 | Cj1351 |
| Chemotaxis | acfB | AfcB, MCP protein required for persistence in the cecum | AL11168.1 | 415144 | 416241 | Cj0448c |
|  | cetA | Campylobacter energy taxis system | AL11168.1 | 1117705 | 1119084 | Cj1190c |
|  | cetB | Campylobacter energy taxis system | AL11168.1 | 1117190 | 1117687 | Cj1189c |
|  | cheA | Chemotaxis protein | AL11168.1 | 260904 | 263213 | Cj0284c |
|  | cheB | Chemotaxis protein | AL11168.1 | 859567 | 860121 | Cj0924c |
|  | cheR | Chemotaxis protein | AL11168.1 | 858763 | 859551 | Cj0923c |
|  | cheV | Chemotaxis protein | AL11168.1 | 263217 | 264173 | Cj0285c |
|  | cheW | Chemotaxis protein | AL11168.1 | 260378 | 260899 | Cj0283c |
|  | CheY | Response regulator controlling flagellar rotation | AL11168.1 | 1050632 | 1051024 | Cj1118c |
|  | luxS | AI-2 biosynthesis enzyme | AL11168.1 | 1127437 | 1127931 | Cj1198 |
|  | tlp1 | Methyl-accepting chemotaxis proteins (MCPs) also called transducer-like proteins | AL11168.1 | 1440883 | 1442985 | Cj1506c |
|  | tlp10 | Methyl-accepting chemotaxis proteins (MCPs) also called transducer-like proteins | AL11168.1 | 23665 | 25443 | Cj0019c |
| Invasion | ciaB | 73-kDa protein involved in adhesion | AL11168.1 | 849834 | 851666 | Cj0914c |
|  | flaC | FlaC protein secreted into the host cells and essential for colonisation and invasion | AL11168.1 | 676227 | 676976 | Cj0720c |
|  | flhA | Component of the flagellar T3SS | AL11168.1 | 817579 | 819753 | Cj0882c |
|  | flhB | Component of the flagellar T3SS | AL11168.1 | 303098 | 304186 | Cj0335 |
|  | fliP | Component of the flagellar T3SS | AL11168.1 | 768029 | 768763 | Cj0820c |
|  | fliQ | Component of the flagellar T3SS | AL11168.1 | 1595774 | 1596043 | Cj1675 |
|  | fliR | Component of the flagellar T3SS | AL11168.1 | 1106317 | 1107084 | Cj1179c |
|  | htrA | chaperone involved in the proper folding of adhesins | AL11168.1 | 1156261 | 1157679 | Cj1228c |
|  | iamA | Invasion associated protein | AL11168.1 | 1572200 | 1572922 | Cj1674 |
| Iron uptake system | ceuE | Lipoprotein involved in iron acquisition | AL11168.1 | 1286672 | 1287664 | Cj1355 |
|  | cfrA | Outer membrane ferric enterobactin FeEnt receptors | AL11168.1 | 705450 | 707540 | Cj0755 |
|  | chuA | Outer membrane receptor for hemin and haemoglobin | AL11168.1 | 1540807 | 1542936 | Cj1614 |
|  | cj0178 | Putative transferring bound iron utilization outer membrane recpetor | AL11168.1 | 173764 | 176031 | Cj0178 |
| Motility | cj1321 | Proteins involved in flagellin O-linked glycosylation | AL11168.1 | 1250852 | 1251394 | Cj1321 |
|  | cj1323 | Proteins involved in flagellin O-linked glycosylation | AL11168.1 | 1251775 | 1252161 | Cj1323 |
|  | cj1324 | Proteins involved in flagellin O-linked glycosylation | AL11168.1 | 1252278 | 1253399 | Cj1324 |
|  | cj1325 | Proteins involved in flagellin O-linked glycosylation | AL11168.1 | 1253417 | 1254092 | Cj1325 |
|  | flgE | Minor hook components | AL11168.1 | 62231 | 63868 | Cj0043 |
|  | flgH | FlgH, L ring in the outer membrane | AL11168.1 | 641102 | 641800 | Cj0687c |
|  | flgI | FlgI, P-ring in the | AL11168.1 | 1398468 | 1399514 | Cj1462 |
|  | fliA | 28 promoter regulates flaA gene expression | AL11168.1 | 75410 | 76126 | Cj0061c |
|  | fliF | FliF, hookebasal body protein | AL11168.1 | 288457 | 290139 | Cj0318 |
|  | fliK | Motility factor, minor hook component | AL11168.1 | 59493 | 61289 | Cj0041 |
|  | fliM | Flagellar motor protein | AL11168.1 | 74331 | 75410 | Cj0060c |
|  | fliY | Flagellar motor protein | AL11168.1 | 73492 | 74334 | Cj0059c |
|  | rpon | 54 promoter regulates flaB gene expression | AL11168.1 | 624138 | 625388 | Cj0670 |
| Multidrug and bile resistance | cmeA | CME efflux pump - periplasmic protein | AL11168.1 | 335718 | 336821 | Cj0367c |
|  | cmeB | CME efflux pump - inner membrane efflux transporter | AL11168.1 | 332596 | 335718 | Cj0366c |
|  | cmeC | CME efflux pump - outer membrane protein | AL11168.1 | 331125 | 332603 | Cj0365c |
|  | cmeR | CmeR, CME efflux pump transcriptional repressor | AL11168.1 | 336916 | 337548 | Cj0368c |
| Stress response | ahpC | Alkyl hydroperoxide reductase | AL11168.1 | 302383 | 302979 | Cj0334 |
|  | cj0012c | Protect against reactive oxygen species | AL11168.1 | 16756 | 17403 | Cj0012c |
|  | cj0020c | Cytochrome c peroxidases | AL11168.1 | 25433 | 26347 | Cj0020c |
|  | Cj0358 | Cytochrome c peroxidases | AL11168.1 | 326024 | 327049 | Cj0358 |
|  | Cj1371 | Protects against reactive oxygen species | AL11168.1 | 1308007 | 1308705 | Cj1371 |
|  | dnaJ | Heat shock protein | AL11168.1 | 1190510 | 1191631 | Cj1260c |
|  | katA | Catalase (convert hydrogen peroxide to water and oxygen) | AL11168.1 | 1322526 | 1323950 | Cj1385 |
|  | spoT | Stringent control | AL11168.1 | 1204596 | 1206791 | Cj1272c |
|  | tpx | Tpx, thiol peroxidase | AL11168.1 | 731405 | 731932 | Cj0779 |
| Toxins | cdtA | Cytolethal distending toxin | AL11168.1 | 90264 | 91070 | Cj0079c |
|  | cdtB | Cytolethal distending toxin | AL11168.1 | 89470 | 90267 | Cj0078c |
|  | cdtC | Cytolethal distending toxin | AL11168.1 | 88890 | 89459 | Cj0077c |
| Type VI secretion system | TagH (VasC) | Type VI secretion protein | JX436460.1 | 1 | 900 |  |
|  | TssM (VasK) | Type VI secretion protein | JX436460.1 | 897 | 4424 |  |
|  | TssD (Hcp) | Type VI secretion protein | JX436460.1 | 4542 | 5057 |  |
|  | TssL (VasF) | Type VI secretion protein | JX436460.1 | 5312 | 6085 |  |
|  | TssK (VasE) | Type VI secretion protein | JX436460.1 | 6082 | 7479 |  |
|  | TssJ (VasD) | Type VI secretion protein | JX436460.1 | 7489 | 7935 |  |
|  | TssA (VasJ) | Type VI secretion protein | JX436460.1 | 8061 | 9308 |  |
|  | TssB (VipA) | Type VI secretion protein | JX436460.1 | 9377 | 9862 |  |
|  | TssC (VipB) | Type VI secretion protein | JX436460.1 | 9864 | 11318 |  |
|  | TssE | Type VI secretion protein | JX436460.1 | 11321 | 11713 |  |
|  | TssF (VasA) | Type VI secretion protein | JX436460.1 | 11710 | 13431 |  |
|  | TssG (VasB) | Type VI secretion protein | JX436460.1 | 13428 | 14336 |  |
|  | TssI (VgrG) | Type VI secretion protein | JX436460.1 | 14486 | 17002 |  |
