# Supplementary material for: Genomic Epidemiology of Campylobacter jejuni Transmission in Israel
Source: Front Microbiol. 2018 Oct 16;9:2432. doi: 10.3389/fmicb.2018.02432 (PMC6198274; doi:10.3389/fmicb.2018.02432)
Supplement: Supplementary file 2 [file Table_2.DOCX]

**SUPPLEMENTARY TABLE 2: LIST OF ISOLATES INCLUDED IN THIS STUDY, METADATA AND SEQUENCE PARAMETERS**

| **Isolate** | **Origin/source** | **Year** | **Ethnicity** | **Age band** | **Sequence type** | **Clonal complex** | **Average read quality** | **Average read coverage** | **N50** | **No. contigs** | **Length** |
| --- | --- | --- | --- | --- | --- | --- | --- | --- | --- | --- | --- |
| 85125 | Human/blood | 2011 | Jewish | >60 | ST-49 | CC-49 | 37 | 210 | 211324 | 17 | 1645592 |
| 74923 | Human/blood | 2010 | Jewish | >60 | ST-49 | CC-49 | 36 | 216 | 228087 | 14 | 1644064 |
| 76957 | Human/blood | 2010 | Jewish | >60 | ST-400 | CC-353 | 36 | 222 | 287232 | 32 | 1740262 |
| 38558 | Human/blood | 2003 | Jewish | >60 | ST-50 | CC-21 | 35 | 265 | 159541 | 28 | 1667014 |
| 69853 | Human/blood | 2009 | Jewish | 0-2 | ST-4057 | CC-574 | 36 | 359 | 225987 | 13 | 1686637 |
| 61601 | Human/blood | 2008 | Non-Jewish | 10-60 | ST-50 | CC-21 | 35 | 338 | 226080 | 19 | 1667118 |
| 73257 | Human/blood | 2010 | Non-Jewish | 10-60 | ST-50 | CC-21 | 34 | 557 | 354067 | 24 | 1634730 |
| 89373 | Human/blood | 2012 | Jewish | 10-60 | ST-49 | CC-49 | 37 | 234 | 229293 | 13 | 1689937 |
| 93312 | Human/blood | 2012 | Jewish | >60 | ST-49 | CC-49 | 36 | 189 | 229298 | 15 | 1690118 |
| 67215 | Human/blood | 2009 | Non-Jewish | 10-60 | ST-572 | CC-206 | 32 | 88 | 90615 | 32 | 1715503 |
| 47468 | Human/blood | 2005 | Non-Jewish | 0-2 | ST-353 | CC-353 | 37 | 309 | 317705 | 15 | 1704280 |
| 47478 | Human/blood | 2005 | Jewish | 2-10 | ST-2122 | CC-353 | 37 | 322 | 174526 | 28 | 1819342 |
| 87217 | Human/blood | 2012 | Non-Jewish | 10-60 | ST-4766 | Unassigned | 36 | 231 | 161836 | 22 | 1831214 |
| 75465 | Human/blood | 2010 | Jewish | 2-10 | ST-4766 | Unassigned | 37 | 305 | 161836 | 24 | 1862534 |
| 73629 | Human/blood | 2010 | Jewish | >60 | ST-1751 | CC-42 | 37 | 245 | 220393 | 24 | 1684505 |
| 39250 | Human/blood | 2003 | Jewish | >60 | ST-42 | CC-42 | 37 | 314 | 187283 | 21 | 1630992 |
| 87698 | Human/blood | 2012 | Jewish | >60 | ST-2337 | CC-353 | 37 | 272 | 183525 | 27 | 1792151 |
| 54567 | Human/blood | 2007 | Non-Jewish | >60 | ST-49 | CC-49 | 37 | 219 | 239578 | 14 | 1618389 |
| 59449 | Human/blood | 2008 | Jewish | >60 | ST-49 | CC-49 | 37 | 204 | 180238 | 16 | 1656118 |
| 83224 | Human/blood | 2011 | Jewish | 0-2 | ST-137 | CC-45 | 37 | 231 | 234518 | 14 | 1604450 |
| 75443 | Human/blood | 2010 | Jewish | 10-60 | ST-6566 | CC-42 | 37 | 232 | 212564 | 17 | 1682511 |
| 69948 | Human/blood | 2009 | Jewish | 10-60 | ST-122 | CC-206 | 37 | 249 | 161847 | 19 | 1647011 |
| 40023 | Human/blood | 2003 | Jewish | 10-60 | ST-5 | CC-353 | 37 | 249 | 286394 | 23 | 1691049 |
| 77138 | Human/blood | 2010 | Jewish | 10-60 | ST-137 | CC-45 | 37 | 147 | 184559 | 27 | 1644619 |
| 86758 | Human/blood | 2012 | Non-Jewish | >60 | ST-7450 | CC-42 | 37 | 199 | 213079 | 16 | 1669922 |
| 43543 | Human/blood | 2004 | Non-Jewish | 2-10 | ST-19 | CC-21 | 37 | 251 | 154055 | 30 | 1617249 |
| 69598 | Human/blood | 2009 | Jewish | >60 | ST-6608 | CC-21 | 37 | 232 | 174605 | 23 | 1709104 |
| 54898 | Human/blood | 2007 | Jewish | 0-2 | ST-467 | CC-49 | 37 | 353 | 289412 | 10 | 1623942 |
| 49329 | Human/blood | 2006 | Jewish | 2-10 | ST-51 | CC-443 | 36 | 254 | 94826 | 40 | 1687706 |
| 68237 | Human/blood | 2009 | Jewish | >60 | ST-50 | CC-21 | 35 | 391 | 189571 | 11 | 1713343 |
| 74087 | Human/blood | 2010 | Jewish | 10-60 | ST-2337 | CC-353 | 38 | 146 | 227594 | 42 | 1783473 |
| 69130 | Human/blood | 2009 | Non-Jewish | 10-60 | ST-460 | CC-460 | 37 | 105 | 148937 | 52 | 1800622 |
| 37123 | Human/blood | 2003 | Jewish | 10-60 | ST-52 | CC-52 | 37 | 440 | 336608 | 42 | 1691764 |
| 55764 | Human/blood | 2007 | Jewish | 10-60 | ST-6608 | CC-21 | 37 | 215 | 189598 | 17 | 1671467 |
| 95201 | Human/blood | 2012 | Non-Jewish | 10-60 | ST-52 | CC-52 | 36 | 213 | 211194 | 18 | 1643992 |
| 78023 | Human/blood | 2011 | Jewish | >60 | ST-6566 | CC-42 | 37 | 250 | 186711 | 14 | 1673501 |
| 76973 | Human/blood | 2010 | Jewish | >60 | ST-6565 | Unassigned | 37 | 298 | 162624 | 23 | 1704627 |
| 73241 | Human/blood | 2010 | Jewish | 10-60 | ST-137 | CC-45 | 37 | 245 | 184244 | 16 | 1645566 |
| 37627 | Human/blood | 2003 | Jewish | >60 | ST-5794 | CC-443 | 37 | 210 | 293803 | 19 | 1640804 |
| 45601 | Human/blood | 2005 | Jewish | >60 | ST-607 | CC-607 | 36 | 300 | 137120 | 106 | 1804969 |
| 67803 | Human/blood | 2009 | Jewish | >60 | ST-658 | CC-658 | 37 | 228 | 323067 | 16 | 1660873 |
| 42948 | Human/blood | 2004 | Jewish | >60 | ST-508 | CC-508 | 36 | 195 | 173471 | 91 | 1754984 |
| 55134 | Human/blood | 2007 | Jewish | >60 | ST-6608 | CC-21 | 36 | 269 | 189597 | 34 | 1737103 |
| 57644 | Human/blood | 2008 | Jewish | 10-60 | ST-49 | CC-49 | 37 | 201 | 240099 | 14 | 1655343 |
| 91626 | Human/blood | 2012 | Jewish | 10-60 | ST-21 | CC-21 | 36 | 263 | 189507 | 37 | 1716065 |
| 39123 | Human/blood | 2003 | Jewish | 0-2 | ST-1923 | CC-257 | 36 | 183 | 178437 | 31 | 1781934 |
| 47753 | Human/blood | 2005 | Non-Jewish | 10-60 | ST-460 | CC-460 | 36 | 331 | 157594 | 96 | 1782741 |
| 93695 | Human/blood | 2012 | Non-Jewish | 0-2 | ST-400 | CC-353 | 37 | 217 | 243620 | 30 | 1744125 |
| 47554 | Human/blood | 2005 | Jewish | 2-10 | ST-354 | CC-354 | 36 | 329 | 355199 | 96 | 1742627 |
| 50118 | Human/blood | 2006 | Jewish | >60 | ST-696 | CC-1332 | 37 | 302 | 306691 | 14 | 1612972 |
| 93225 | Human/blood | 2012 | Non-Jewish | 0-2 | ST-7400 | Unassigned | 37 | 225 | 243468 | 64 | 1800641 |
| 56786 | Human/stool | 2008 | Jewish | 2-10 | ST-21 | CC-21 | 36 | 306 | 227540 | 15 | 1734177 |
| 51955 | Human/stool | 2006 | Jewish | 0-2 | ST-21 | CC-21 | 37 | 286 | 189507 | 17 | 1706159 |
| 72503 | Human/stool | 2010 | Jewish | 2-10 | ST-21 | CC-21 | 36 | 447 | 176346 | 21 | 1706294 |
| 67651 | Human/stool | 2009 | Jewish | 2-10 | ST-21 | CC-21 | 37 | 235 | 176346 | 20 | 1706523 |
| 57111 | Human/stool | 2008 | Jewish | 0-2 | ST-883 | CC-21 | 36 | 285 | 295479 | 8 | 1666078 |
| 45803 | Human/stool | 2005 | Non-Jewish | 2-10 | ST-572 | CC-206 | 32 | 64 | 37102 | 87 | 1712906 |
| 57428 | Human/stool | 2008 | Non-Jewish | 2-10 | ST-257 | CC-257 | 35 | 295 | 332987 | 9 | 1687504 |
| 48945 | Human/stool | 2006 | Jewish | 10-60 | ST-572 | CC-206 | 32 | 73 | 24637 | 120 | 1756660 |
| 54906 | Human/stool | 2007 | Jewish | >60 | ST-257 | CC-257 | 33 | 203 | 331897 | 8 | 1693238 |
| 64289 | Human/stool | 2009 | Jewish | 2-10 | ST-883 | CC-21 | 36 | 220 | 295495 | 12 | 1626741 |
| 50288 | Human/stool | 2006 | Jewish | 2-10 | ST-50 | CC-21 | 35 | 450 | 269854 | 20 | 1713300 |
| 60441 | Human/stool | 2008 | Non-Jewish | 0-2 | ST-50 | CC-21 | 35 | 436 | 261818 | 13 | 1712110 |
| 52104 | Human/stool | 2006 | Jewish | 0-2 | ST-1359 | CC-21 | 34 | 310 | 221569 | 14 | 1754553 |
| 62690 | Human/stool | 2008 | Jewish | 2-10 | ST-572 | CC-206 | 32 | 87 | 63737 | 54 | 1648745 |
| 56547 | Human/stool | 2007 | Jewish | 10-60 | ST-572 | CC-206 | 32 | 92 | 53350 | 57 | 1649804 |
| 54362 | Human/stool | 2007 | Non-Jewish | 2-10 | ST-572 | CC-206 | 32 | 66 | 56282 | 64 | 1676976 |
| 70958 | Human/stool | 2010 | Jewish | 0-2 | ST-257 | CC-257 | 35 | 273 | 333645 | 11 | 1692325 |
| 87116 | Human/stool | 2012 | Jewish | 10-60 | ST-50 | CC-21 | 35 | 424 | 1026943 | 14 | 1630797 |
| 52105 | Human/stool | 2006 | Non-Jewish | 0-2 | ST-1359 | CC-21 | 35 | 299 | 221569 | 18 | 1754403 |
| 49369 | Human/stool | 2006 | Jewish | 10-60 | ST-400 | CC-353 | 36 | 264 | 104698 | 790 | 2156620 |
| 56969 | Human/stool | 2008 | Non-Jewish | 0-2 | ST-1359 | CC-21 | 35 | 284 | 188275 | 18 | 1747088 |
| 44423 | Human/stool | 2005 | Non-Jewish | 0-2 | ST-50 | CC-21 | 34 | 264 | 875559 | 11 | 1714399 |
| 92306 | Human/stool | 2012 | Jewish | 2-10 | ST-572 | CC-206 | 32 | 79 | 34436 | 87 | 1676188 |
| 79390 | Human/stool | 2011 | Jewish | >60 | ST-572 | CC-206 | 32 | 65 | 41610 | 68 | 1642967 |
| 56986 | Human/stool | 2008 | Jewish | 2-1 | ST-50 | CC-21 | 35 | 329 | 294215 | 15 | 1663057 |
| 52608 | Human/stool | 2007 | Jewish | 0-2 | ST-1359 | CC-21 | 34 | 307 | 150135 | 21 | 1777431 |
| 54927 | Human/stool | 2007 | Jewish | 0-2 | ST-1359 | CC-21 | 35 | 343 | 150135 | 28 | 1779356 |
| 41354 | Human/stool | 2004 | Non-Jewish | 2-10 | ST-257 | CC-257 | 31 | 659 | 293759 | 9 | 1654797 |
| 49861 | Human/stool | 2006 | Jewish | 10-60 | ST-257 | CC-257 | 34 | 238 | 293762 | 9 | 1654320 |
| 49698 | Human/stool | 2006 | Jewish | 10-60 | ST-257 | CC-257 | 35 | 297 | 293762 | 9 | 1654316 |
| 41150 | Human/stool | 2004 | Jewish | 10-60 | ST-883 | CC-21 | 36 | 209 | 154624 | 77 | 1653616 |
| 73287 | Human/stool | 2010 | Jewish | 0-2 | ST-257 | CC-257 | 34 | 248 | 869159 | 8 | 1730677 |
| 79685 | Human/stool | 2011 | Jewish | 10-60 | ST-572 | CC-206 | 30 | 71 | 68775 | 51 | 1639590 |
| 50047 | Human/stool | 2006 | Non-Jewish | >60 | ST-1359 | CC-21 | 34 | 297 | 188277 | 21 | 1733961 |
| 83986 | Human/stool | 2011 | Non-Jewish | 10-60 | ST-4057 | CC-574 | 36 | 211 | 225986 | 16 | 1749019 |
| 50096 | Human/stool | 2006 | Jewish | >60 | ST-21 | CC-21 | 37 | 321 | 189522 | 21 | 1639859 |
| 42782 | Human/stool | 2004 | Jewish | >60 | ST-50 | CC-21 | 35 | 345 | 332375 | 15 | 1662971 |
| 43912 | Human/stool | 2004 | Non-Jewish | 10-60 | ST-50 | CC-21 | 35 | 349 | 294214 | 15 | 1662195 |
| 60575 | Human/stool | 2008 | Jewish | 0-2 | ST-257 | CC-257 | 35 | 258 | 242656 | 22 | 1724867 |
| 45214 | Human/stool | 2005 | Jewish | 2-10 | ST-50 | CC-21 | 33 | 293 | 294214 | 15 | 1662171 |
| 56623 | Human/stool | 2007 | Jewish | 10-60 | ST-257 | CC-257 | 35 | 319 | 332987 | 8 | 1688087 |
| 77938 | Human/stool | 2011 | Jewish | >60 | ST-883 | CC-21 | 36 | 209 | 295574 | 11 | 1656577 |
| 49714 | Human/stool | 2006 | Non-Jewish | 10-60 | ST-257 | CC-257 | 35 | 508 | 332987 | 18 | 1699162 |
| 37086 | Human/stool | 2003 | Non-Jewish | 0-2 | ST-4057 | CC-574 | 36 | 240 | 186665 | 37 | 1695324 |
| 49908 | Human/stool | 2006 | Non-Jewish | 0-2 | ST-21 | CC-21 | 37 | 322 | 175605 | 25 | 1639446 |
| 77305 | Human/stool | 2010 | Non-Jewish | 0-2 | ST-257 | CC-257 | 35 | 248 | 333157 | 9 | 1653864 |
| 57740 | Human/stool | 2008 | Non-Jewish | 0-2 | ST-21 | CC-21 | 36 | 206 | 189525 | 16 | 1639298 |
| 48594 | Human/stool | 2006 | Jewish | 2-10 | ST-460 | CC-460 | 37 | 345 | 175566 | 70 | 1770157 |
| 67665 | Human/stool | 2009 | Jewish | 2-10 | ST-4057 | CC-574 | 37 | 297 | 176074 | 18 | 1686653 |
| 52041 | Human/stool | 2006 | Jewish | 2-10 | ST-21 | CC-21 | 36 | 316 | 189507 | 21 | 1708027 |
| 51319 | Human/stool | 2006 | Jewish | 0-2 | ST-21 | CC-21 | 36 | 264 | 189523 | 18 | 1638367 |
| 49563 | Human/stool | 2006 | Jewish | 2-10 | ST-1359 | CC-21 | 35 | 307 | 188275 | 18 | 1734510 |
| 77544 | Human/stool | 2010 | Jewish | 0-2 | ST-257 | CC-257 | 35 | 274 | 326747 | 12 | 1653888 |
| 48511 | Human/stool | 2006 | Jewish | 0-2 | ST-1359 | CC-21 | 35 | 341 | 188275 | 22 | 1733038 |
| 75914 | Human/stool | 2010 | Jewish | >60 | ST-257 | CC-257 | 35 | 303 | 324722 | 10 | 1679647 |
| 77627 | Human/stool | 2010 | Jewish | 0-2 | ST-21 | CC-21 | 36 | 275 | 189508 | 19 | 1708032 |
| 56971 | Human/stool | 2008 | Jewish | 2-10 | ST-257 | CC-257 | 34 | 217 | 332988 | 7 | 1650693 |
| 54988 | Human/stool | 2007 | Jewish | 0-2 | ST-257 | CC-257 | 35 | 486 | 332990 | 12 | 1650077 |
| 72945 | Human/stool | 2010 | Jewish | 10-60 | ST-572 | CC-206 | 33 | 77 | 65691 | 43 | 1644146 |
| 44837 | Human/stool | 2005 | Jewish | 0-2 | ST-50 | CC-21 | 35 | 306 | 159540 | 32 | 1686076 |
| 56957 | Human/stool | 2008 | Jewish | >60 | ST-50 | CC-21 | 34 | 238 | 197929 | 18 | 1663083 |
| 48778 | Human/stool | 2006 | Jewish | 0-2 | ST-460 | CC-460 | 36 | 161 | 89311 | 89 | 1809292 |
| 51385 | Human/stool | 2006 | Non-Jewish | 10-60 | ST-257 | CC-257 | 35 | 287 | 293759 | 10 | 1656449 |
| 56172 | Human/stool | 2007 | Jewish | >60 | ST-257 | CC-257 | 35 | 620 | 332988 | 8 | 1650267 |
| 78555 | Human/stool | 2011 | Jewish | 10-60 | ST-572 | CC-206 | 33 | 78 | 84468 | 42 | 1679610 |
| 87566 | Human/stool | 2012 | Non-Jewish | 0-2 | ST-257 | CC-257 | 35 | 295 | 332988 | 12 | 1649934 |
| 86673 | Human/stool | 2011 | Jewish | 10-60 | ST-257 | CC-257 | 35 | 338 | 332987 | 12 | 1650077 |
| 47845 | Human/stool | 2005 | Jewish | 10-60 | ST-4057 | CC-574 | 36 | 203 | 154586 | 24 | 1722302 |
| 75710 | Human/stool | 2010 | Jewish | 0-2 | ST-4057 | CC-574 | 36 | 302 | 128624 | 24 | 1722477 |
| 95023 | Human/stool | 2012 | Jewish | 10-60 | ST-257 | CC-257 | 35 | 330 | 331136 | 7 | 1651696 |
| 52737 | Human/stool | 2007 | Non-Jewish | 0-2 | ST-4057 | CC-574 | 36 | 280 | 225987 | 15 | 1686742 |
| 79551 | Human/stool | 2011 | Non-Jewish | 0-2 | ST-572 | CC-206 | 32 | 59 | 75402 | 37 | 1679602 |
| 42352 | Human/stool | 2004 | Non-Jewish | 10-60 | ST-257 | CC-257 | 32 | 662 | 293669 | 8 | 1609535 |
| 60447 | Human/stool | 2008 | Jewish | 2-10 | ST-257 | CC-257 | 34 | 270 | 287071 | 12 | 1614191 |
| 49957 | Human/stool | 2006 | Jewish | 10-60 | ST-50 | CC-21 | 35 | 407 | 226042 | 21 | 1667350 |
| 49626 | Human/stool | 2006 | Jewish | 2-10 | ST-50 | CC-21 | 32 | 552 | 226042 | 21 | 1667133 |
| 92157 | Human/stool | 2012 | Jewish | 0-2 | ST-572 | CC-206 | 32 | 66 | 134116 | 21 | 1680395 |
| 50446 | Human/stool | 2006 | Non-Jewish | 0-2 | ST-1359 | CC-21 | 34 | 425 | 188276 | 15 | 1676160 |
| 72050 | Human/stool | 2010 | Jewish | 2-10 | ST-4057 | CC-574 | 36 | 333 | 225985 | 18 | 1724761 |
| 67999 | Human/stool | 2009 | Jewish | 0-2 | ST-50 | CC-21 | 35 | 387 | 159970 | 24 | 1633556 |
| 88808 | Human/stool | 2012 | Jewish | 10-60 | ST-4057 | CC-574 | 37 | 287 | 125298 | 46 | 1730075 |
| 77734 | Human/stool | 2010 | Jewish | 2-10 | ST-883 | CC-21 | 36 | 284 | 295529 | 13 | 1657876 |
| 57769 | Human/stool | 2008 | Jewish | 2-10 | ST-50 | CC-21 | 31 | 558 | 199398 | 17 | 1751479 |
| 67799 | Human/stool | 2009 | Jewish | 10-60 | ST-50 | CC-21 | 35 | 344 | 159979 | 22 | 1633304 |
| 43197 | Human/stool | 2004 | Jewish | 0-2 | ST-50 | CC-21 | 35 | 321 | 159540 | 22 | 1620905 |
| 42001 | Human/stool | 2004 | Jewish | 2-10 | ST-883 | CC-21 | 36 | 345 | 295479 | 9 | 1627665 |
| 54379 | Human/stool | 2007 | Jewish | 10-60 | ST-4057 | CC-574 | 37 | 428 | 176075 | 17 | 1685761 |
| 73110 | Human/stool | 2010 | Non-Jewish | 0-2 | ST-4057 | CC-574 | 36 | 281 | 225986 | 13 | 1685850 |
| 52691 | Human/stool | 2007 | Jewish | 0-2 | ST-21 | CC-21 | 36 | 282 | 189523 | 22 | 1642025 |
| 37046 | Human/stool | 2003 | Jewish | 2-10 | ST-572 | CC-206 | 32 | 61 | 108785 | 39 | 1769899 |
| 60661 | Human/stool | 2008 | Jewish | 2-10 | ST-883 | CC-21 | 37 | 313 | 399955 | 77 | 1692357 |
| 87629 | Human/stool | 2012 | Jewish | 0-2 | ST-50 | CC-21 | 35 | 334 | 159979 | 24 | 1630357 |
| 60663 | Human/stool | 2008 | Jewish | >60 | ST-21 | CC-21 | 36 | 207 | 227773 | 19 | 1744462 |
| 41197 | Human/stool | 2004 | Jewish | 10-60 | ST-572 | CC-206 | 32 | 58 | 29904 | 130 | 1831069 |
| 47263 | Human/stool | 2005 | Jewish | 0-2 | ST-6874 | Unassigned | 36 | 245 | 169969 | 70 | 1860974 |
| 75417 | Human/stool | 2010 | Jewish | 0-2 | ST-50 | CC-21 | 35 | 445 | 1020110 | 23 | 1627581 |
| 44206 | Human/stool | 2004 | Non-Jewish | 0-2 | ST-50 | CC-21 | 35 | 332 | 1031976 | 12 | 1625121 |
| 77569 | Human/stool | 2010 | Jewish | 10-60 | ST-572 | CC-206 | 32 | 79 | 71105 | 49 | 1646192 |
| 48756 | Human/stool | 2006 | Jewish | 0-2 | ST-572 | CC-206 | 33 | 71 | 56037 | 50 | 1640611 |
| 52046 | Human/stool | 2006 | Jewish | >60 | ST-572 | CC-206 | 32 | 68 | 59025 | 58 | 1644227 |
| 60230 | Human/stool | 2008 | Jewish | 10-60 | ST-572 | CC-206 | 32 | 81 | 28550 | 93 | 1689574 |
| 57254 | Human/stool | 2008 | Jewish | >60 | ST-572 | CC-206 | 32 | 82 | 62587 | 43 | 1646797 |
| 63874 | Human/stool | 2009 | Jewish | 2-10 | ST-572 | CC-206 | 33 | 86 | 98328 | 35 | 1647625 |
| 56974 | Human/stool | 2008 | Jewish | 0-2 | ST-572 | CC-206 | 32 | 64 | 44467 | 86 | 1727670 |
| 43665 | Human/stool | 2004 | Non-Jewish | 0-2 | ST-1359 | CC-21 | 35 | 274 | 188275 | 61 | 1814215 |
| 72450 | Human/stool | 2010 | Jewish | 2-10 | ST-883 | CC-21 | 36 | 254 | 295434 | 9 | 1626476 |
| 35992 | Human/stool | 2003 | Jewish | 2-10 | ST-572 | CC-206 | 32 | 65 | 42260 | 82 | 1715718 |
| 35872 | Human/stool | 2003 | Non-Jewish | 0-2 | ST-7547 | CC-48 | 36 | 376 | 191392 | 14 | 1643335 |
| 62686 | Human/stool | 2008 | Jewish | 0-2 | ST-572 | CC-206 | 32 | 78 | 51319 | 58 | 1715959 |
| 76636 | Human/stool | 2010 | Jewish | 10-60 | ST-572 | CC-206 | 32 | 81 | 98740 | 40 | 1641654 |
| 49467 | Human/stool | 2006 | Jewish | 2-10 | ST-468 | Unassigned | 36 | 582 | 144428 | 41 | 1634634 |
| 35986 | Human/stool | 2003 | Jewish | 2-10 | ST-6627 | CC-21 | 36 | 135 | 295459 | 12 | 1672609 |
| 44845 | Human/stool | 2005 | Non-Jewish | 0-2 | ST-50 | CC-21 | 35 | 346 | 159539 | 22 | 1624166 |
| 42548 | Human/stool | 2004 | Jewish | 10-60 | ST-50 | CC-21 | 35 | 249 | 188295 | 17 | 1698688 |
| 45955 | Human/stool | 2005 | Non-Jewish | 2-10 | ST-6566 | CC-42 | 36 | 250 | 224834 | 12 | 1673837 |
| 48593 | Human/stool | 2006 | Non-Jewish | 0-2 | ST-400 | CC-353 | 36 | 226 | 176392 | 41 | 1770649 |
| 79401 | Human/stool | 2011 | Jewish | 10-60 | ST-50 | CC-21 | 34 | 548 | 294448 | 24 | 1625004 |
| 78761 | Human/stool | 2011 | Jewish | 2-10 | ST-257 | CC-257 | 35 | 255 | 333040 | 12 | 1698218 |
| 60665 | Human/stool | 2008 | Jewish | 10-60 | ST-50 | CC-21 | 35 | 355 | 188296 | 34 | 1697338 |
| 47573 | Human/stool | 2005 | Jewish | 10-60 | ST-2927 | CC-607 | 36 | 277 | 213044 | 124 | 1832958 |
| 38345 | Human/stool | 2003 | Jewish | >60 | ST-42 | CC-42 | 37 | 293 | 186579 | 90 | 1710504 |
| 42230 | Human/stool | 2004 | Jewish | 2-10 | ST-190 | CC-21 | 36 | 283 | 218830 | 58 | 1720284 |
| 41254 | Human/stool | 2004 | Jewish | 0-2 | ST-257 | CC-257 | 32 | 582 | 289204 | 14 | 1661504 |
| 43400 | Human/stool | 2004 | Jewish | 2-10 | ST-257 | CC-257 | 34 | 257 | 295532 | 12 | 1662282 |
| 48795 | Human/stool | 2006 | Jewish | 10-60 | ST-6977 | CC-446 | 36 | 286 | 144137 | 35 | 1726156 |
| 66320 | Human/stool | 2009 | Jewish | 0-2 | ST-257 | CC-257 | 35 | 290 | 287138 | 10 | 1614144 |
| 42276 | Human/stool | 2004 | Jewish | 2-10 | ST-122 | CC-206 | 36 | 299 | 106652 | 86 | 1715606 |
| 55519 | Human/stool | 2007 | Jewish | >60 | ST-50 | CC-21 | 35 | 323 | 159541 | 18 | 1630026 |
| 49727 | Human/stool | 2006 | Jewish | 2-10 | ST-6608 | CC-21 | 36 | 305 | 189597 | 18 | 1672248 |
| 43431 | Human/stool | 2004 | Jewish | 0-2 | ST-905 | Unassigned | 36 | 213 | 276020 | 36 | 1597559 |
| 57129 | Human/stool | 2008 | Jewish | >60 | ST-50 | CC-21 | 34 | 279 | 332764 | 17 | 1663724 |
| 46108 | Human/stool | 2005 | Jewish | 10-60 | ST-52 | CC-52 | 37 | 88 | 181537 | 27 | 1605217 |
| 56972 | Human/stool | 2008 | Jewish | 10-60 | ST-572 | CC-206 | 33 | 90 | 51559 | 73 | 1643969 |
| 49205 | Human/stool | 2006 | Jewish | 10-60 | ST-49 | CC-49 | 37 | 294 | 238236 | 17 | 1619173 |
| 43019 | Human/stool | 2004 | Non-Jewish | 0-2 | ST-52 | CC-52 | 36 | 112 | 107907 | 43 | 1612939 |
| 70421 | Human/stool | 2010 | Jewish | 0-2 | ST-50 | CC-21 | 34 | 393 | 159540 | 33 | 1694389 |
| 41988 | Human/stool | 2004 | Non-Jewish | 2-10 | ST-572 | CC-206 | 33 | 64 | 53207 | 57 | 1642716 |
| 53818 | Human/stool | 2007 | Jewish | 0-2 | ST-1359 | CC-21 | 35 | 376 | 188279 | 30 | 1792114 |
| 56981 | Human/stool | 2008 | Non-Jewish | 0-2 | ST-1359 | CC-21 | 35 | 257 | 189323 | 25 | 1790921 |
| 49264 | Human/stool | 2006 | Jewish | 0-2 | ST-50 | CC-21 | 35 | 313 | 154025 | 27 | 1752640 |
| 89880 | Human/stool | 2012 | Jewish | 2-10 | ST-572 | CC-206 | 32 | 54 | 96883 | 52 | 1651479 |
| 43803 | Human/stool | 2004 | Non-Jewish | 0-2 | ST-1359 | CC-21 | 33 | 239 | 189175 | 22 | 1770772 |
| 67702 | Human/stool | 2009 | Jewish | 10-60 | ST-4057 | CC-574 | 36 | 365 | 225986 | 14 | 1685888 |
| 45882 | Human/stool | 2005 | Jewish | 0-2 | ST-1359 | CC-21 | 34 | 260 | 188274 | 32 | 1791582 |
| 54574 | Human/stool | 2007 | Jewish | 2-10 | ST-50 | CC-21 | 35 | 372 | 154057 | 28 | 1695496 |
| 64436 | Human/stool | 2009 | Jewish | 0-2 | ST-572 | CC-206 | 32 | 57 | 63666 | 59 | 1650449 |
| 67005 | Human/stool | 2009 | Non-Jewish | 0-2 | ST-572 | CC-206 | 32 | 76 | 111987 | 35 | 1650281 |
| 41523 | Human/stool | 2004 | Non-Jewish | 2-10 | ST-5285 | CC-607 | 36 | 215 | 183789 | 175 | 1909549 |
| 90475 | Human/stool | 2012 | Jewish | 0-2 | ST-1359 | CC-21 | 35 | 255 | 177072 | 36 | 1848241 |
| 38194 | Human/stool | 2003 | Non-Jewish | 0-2 | ST-572 | CC-206 | 32 | 61 | 39249 | 87 | 1682349 |
| 43379 | Human/stool | 2004 | Jewish | 2-10 | ST-50 | CC-21 | 35 | 471 | 154047 | 45 | 1695442 |
| 42353 | Human/stool | 2004 | Jewish | 2-10 | ST-50 | CC-21 | 35 | 257 | 294216 | 17 | 1664613 |
| 35998 | Human/stool | 2003 | Jewish | 2-10 | ST-883 | CC-21 | 37 | 356 | 217014 | 41 | 1873616 |
| 48396 | Human/stool | 2006 | Non-Jewish | 10-60 | ST-400 | CC-353 | 36 | 286 | 183199 | 34 | 1741190 |
| 45911 | Human/stool | 2005 | Jewish | 2-10 | ST-50 | CC-21 | 35 | 384 | 188347 | 22 | 1656909 |
| 47784 | Human/stool | 2005 | Jewish | 10-60 | ST-6565 | Unassigned | 36 | 286 | 146546 | 71 | 1724388 |
| 38482 | Human/stool | 2003 | Non-Jewish | 0-2 | ST-4057 | CC-574 | 36 | 202 | 113204 | 50 | 1768871 |
| 86850 | Human/stool | 2012 | Jewish | >60 | ST-257 | CC-257 | 35 | 331 | 332983 | 12 | 1650077 |
| 42802 | Human/stool | 2004 | Jewish | 0-2 | ST-3269 | CC-446 | 37 | 380 | 176618 | 39 | 1731508 |
| 49427 | Human/stool | 2006 | Jewish | 0-2 | ST-6565 | Unassigned | 36 | 252 | 99265 | 91 | 1728140 |
| 48779 | Human/stool | 2006 | Non-Jewish | 2-10 | ST-1474 | CC-353 | 37 | 330 | 121541 | 70 | 1731661 |
| 77409 | Human/stool | 2010 | Jewish | 0-2 | ST-883 | CC-21 | 36 | 238 | 222398 | 42 | 1638480 |
| 43020 | Human/stool | 2004 | Non-Jewish | 10-60 | ST-49 | CC-49 | 36 | 230 | 194512 | 23 | 1657075 |
| 41471 | Human/stool | 2004 | Non-Jewish | >60 | ST-883 | CC-21 | 37 | 350 | 228631 | 57 | 1645312 |
| 45737 | Human/stool | 2005 | Jewish | 2-10 | ST-6713 | CC-353 | 36 | 283 | 565009 | 24 | 1712173 |
| 76564 | Human/stool | 2010 | Jewish | 0-2 | ST-883 | CC-21 | 36 | 296 | 1028070 | 12 | 1631256 |
| 44153 | Human/stool | 2004 | Non-Jewish | 2-10 | ST-1359 | CC-21 | 34 | 222 | 188276 | 38 | 1796454 |
| 50400 | Human/stool | 2006 | Jewish | 2-10 | ST-1359 | CC-21 | 35 | 459 | 188276 | 18 | 1785426 |
| 44529 | Human/stool | 2005 | Jewish | 0-2 | ST-881 | Unassigned | 36 | 351 | 261459 | 54 | 1714108 |
| 88796 | Human/stool | 2012 | Jewish | 2-10 | ST-4057 | CC-574 | 37 | 288 | 251932 | 37 | 1742346 |
| 86664 | Human/stool | 2012 | Jewish | 0-2 | ST-50 | CC-21 | 34 | 410 | 255512 | 21 | 1728303 |
| 43962 | Human/stool | 2004 | Jewish | 0-2 | ST-50 | CC-21 | 34 | 217 | 226045 | 14 | 1669862 |
| 37059 | Human/stool | 2003 | Jewish | 10-60 | ST-6978 | CC-21 | 36 | 110 | 175798 | 27 | 1737565 |
| 49361 | Human/stool | 2006 | Jewish | 0-2 | ST-460 | CC-460 | 36 | 314 | 301187 | 47 | 1772762 |
| 67588 | Human/stool | 2009 | Jewish | 0-2 | ST-883 | CC-21 | 37 | 316 | 295387 | 27 | 1705279 |
| 49472 | Human/stool | 2006 | Non-Jewish | 2-10 | ST-6695 | Unassigned | 37 | 423 | 187256 | 17 | 1632567 |
| 76262 | Human/stool | 2010 | Jewish | 2-10 | ST-883 | CC-21 | 37 | 274 | 219354 | 75 | 1653499 |
| 43841 | Human/stool | 2004 | Non-Jewish | 10-60 | ST-354 | CC-354 | 36 | 225 | 379987 | 17 | 1706917 |
| 67219 | Human/stool | 2009 | Jewish | 0-2 | ST-1359 | CC-21 | 35 | 279 | 188277 | 18 | 1791022 |
| 75686 | Human/stool | 2010 | Jewish | 10-60 | ST-883 | CC-21 | 36 | 201 | 223027 | 17 | 1704268 |
| 52441 | Human/stool | 2007 | Jewish | 10-60 | ST-1359 | CC-21 | 35 | 403 | 188310 | 17 | 1808166 |
| 90249 | Human/stool | 2012 | Jewish | 10-60 | ST-50 | CC-21 | 35 | 319 | 189570 | 11 | 1713280 |
| 47379 | Human/stool | 2005 | Jewish | 0-2 | ST-1962 | Unassigned | 36 | 243 | 207044 | 20 | 1680461 |
| 48630 | Human/stool | 2006 | Jewish | 0-2 | ST-6695 | Unassigned | 36 | 291 | 105066 | 164 | 1685644 |
| 77014 | Human/stool | 2010 | Non-Jewish | >60 | ST-21 | CC-21 | 37 | 444 | 180419 | 23 | 1803186 |
| 93196 | Human/stool | 2012 | Jewish | >60 | ST-572 | CC-206 | 32 | 68 | 90256 | 43 | 1647092 |
| 48617 | Human/stool | 2006 | Jewish | >60 | ST-6566 | CC-42 | 36 | 172 | 212585 | 12 | 1676091 |
| 49689 | Human/stool | 2006 | Jewish | >60 | ST-354 | CC-354 | 36 | 336 | 451913 | 13 | 1705969 |
| 210p | Poultry | 2008 | - | - | ST-1359 | CC-21 | 34 | 263 | 188276 | 25 | 1794559 |
| 211p | Poultry | 2008 | - | - | ST-1359 | CC-21 | 35 | 326 | 188276 | 28 | 1794386 |
| 56 | Poultry | 2014 | - | - | ST-572 | CC-206 | 32 | 74 | 197900 | 32 | 1651210 |
| 140 | Poultry | 2014 | - | - | ST-257 | CC-257 | 32 | 600 | 332176 | 8 | 1651584 |
| 189 | Poultry | 2014 | - | - | ST-21 | CC-21 | 36 | 186 | 176346 | 15 | 1705997 |
| 68a | Poultry | 2014 | - | - | ST-50 | CC-21 | 34 | 319 | 189570 | 14 | 1718151 |
| 95p | Poultry | 2008 | - | - | ST-4057 | CC-574 | 37 | 267 | 149708 | 28 | 1720285 |
| 94 | Poultry | 2014 | - | - | ST-50 | CC-21 | 35 | 262 | 154047 | 23 | 1628250 |
| 40 | Poultry | 2014 | - | - | ST-572 | CC-206 | 32 | 561 | 191332 | 16 | 1641898 |
| 5 | Poultry | 2013 | - | - | ST-257 | CC-257 | 32 | 253 | 293695 | 8 | 1617111 |
| 28 | Poultry | 2014 | - | - | ST-257 | CC-257 | 33 | 583 | 287274 | 9 | 1617203 |
| 36 | Poultry | 2014 | - | - | ST-572 | CC-206 | 32 | 75 | 62737 | 100 | 1683662 |
| 169p | Poultry | 2008 | - | - | ST-257 | CC-257 | 35 | 336 | 242655 | 21 | 1723922 |
| 119p | Poultry | 2008 | - | - | ST-4057 | CC-574 | 36 | 268 | 151598 | 59 | 1737856 |
| 9lul | Poultry | 2015 | - | - | ST-4057 | CC-574 | 37 | 275 | 145278 | 21 | 1720588 |
| 151 | Poultry | 2014 | - | - | ST-883 | CC-21 | 36 | 217 | 366268 | 33 | 1643086 |
| 208p | Poultry | 2008 | - | - | ST-21 | CC-21 | 36 | 234 | 176346 | 276 | 1870089 |
| 31b | Poultry | 2014 | - | - | ST-50 | CC-21 | 35 | 325 | 446012 | 20 | 1679431 |
| 29 | Poultry | 2014 | - | - | ST-21 | CC-21 | 36 | 233 | 176346 | 15 | 1704225 |
| 191p | Poultry | 2008 | - | - | ST-257 | CC-257 | 35 | 271 | 332988 | 14 | 1725104 |
| 6 | Poultry | 2013 | - | - | ST-257 | CC-257 | 32 | 540 | 293691 | 8 | 1617105 |
| 45c | Bovine | 2008 | - | - | ST-572 | CC-206 | 33 | 90 | 89858 | 37 | 1644038 |
| 78c | Bovine | 2008 | - | - | ST-572 | CC-206 | 32 | 387 | 153502 | 18 | 1727129 |
| 9c | Bovine | 2008 | - | - | ST-21 | CC-21 | 37 | 309 | 135191 | 34 | 1707558 |
