# Supplementary material for: Genomic Epidemiology of Campylobacter jejuni Transmission in Israel
Source: Front Microbiol. 2018 Oct 16;9:2432. doi: 10.3389/fmicb.2018.02432 (PMC6198274; doi:10.3389/fmicb.2018.02432)
Supplement: Supplementary file 3 [file Image_1.pdf]

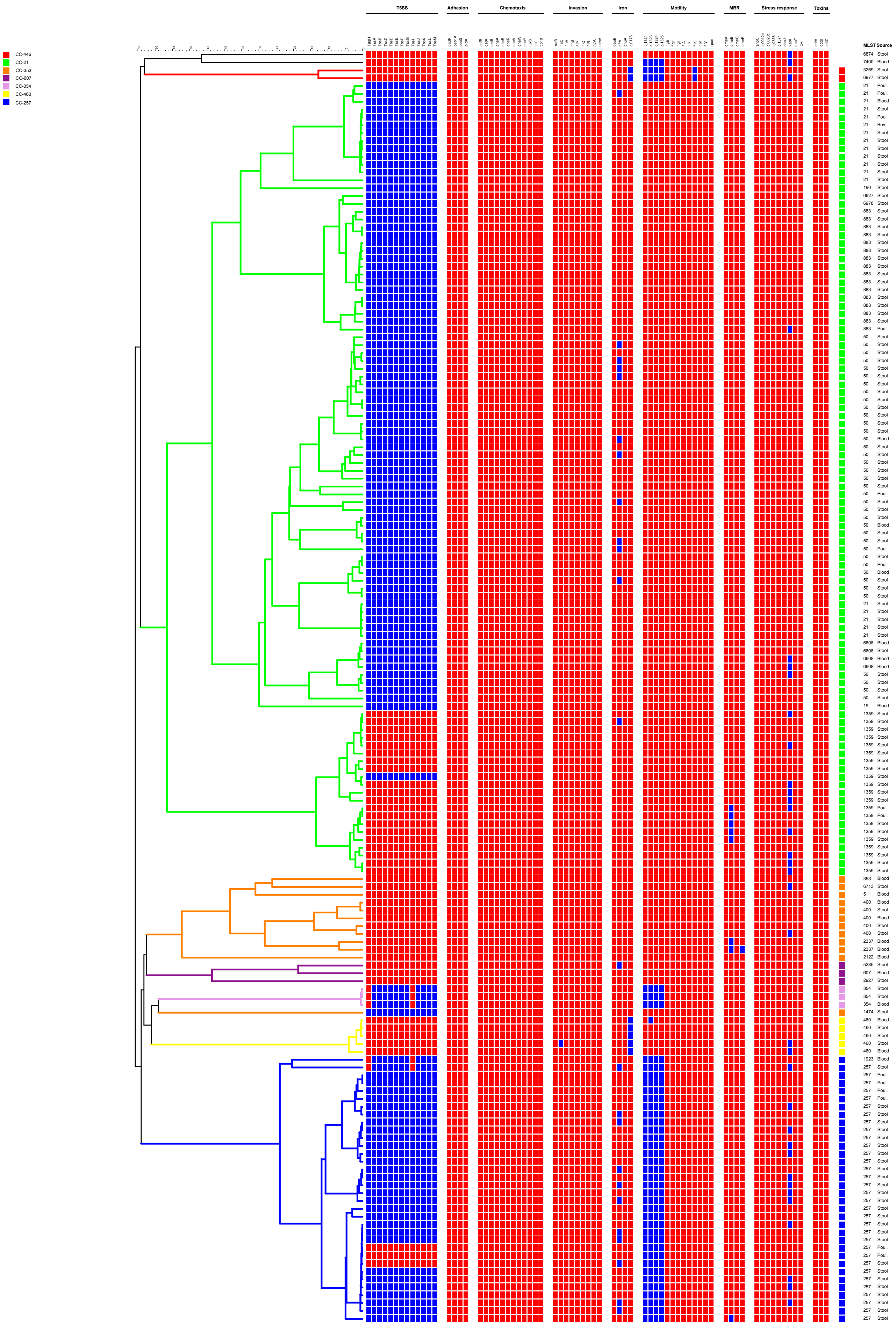

CC-446
CC-21
CC-353
CC-607
CC-354
CC-460
CC-257
T6SS
Adhesion
Chemotaxis
Invasion
Iron
Motility
MBR
Stress response
Toxins
TagH TssA TssB TssC TssD TssE TssF TssG TssI TssJ TssK TssL TssM
cadF peb1A peb3 pldA
acfB cetA cetB cheA cheB cheR cheV cheW cheY luxS tlp1 tlp10
ciaB flaC flhA flhB fliP fliQ fliR htrA iamA
ceuE cfrA chuA cj0178
cj1321 cj1323 cj1324 cj1325 flgE flgH flgI fliA fliF fliK fliM fliY rpon
cmeA cmeB cmeC cmeR
ahpC cj0012c cj0020c cj0358 cj1371 dnaJ katA spoT tpx
cdtA cdtB cdtC
MLST Source
6874 Stool
7400 Blood
3269 Stool
6977 Stool
21 Poul.
21 Poul.
21 Blood
21 Stool
21 Poul.
21 Bov.
21 Stool
21 Stool
21 Stool
21 Stool
21 Stool
21 Stool
21 Stool
190 Stool
6627 Stool
6978 Stool
883 Stool
883 Stool
883 Stool
883 Stool
883 Stool
883 Stool
883 Stool
883 Stool
883 Stool
883 Stool
883 Stool
883 Stool
883 Stool
883 Stool
883 Stool
883 Poul.
50 Stool
50 Stool
50 Stool
50 Stool
50 Stool
50 Stool
50 Stool
50 Stool
50 Stool
50 Stool
50 Stool
50 Stool
50 Stool
50 Blood
50 Stool
50 Stool
50 Stool
50 Stool
50 Stool
50 Stool
50 Poul.
50 Stool
50 Stool
50 Stool
50 Blood
50 Stool
50 Stool
50 Poul.
50 Stool
50 Poul.
50 Blood
50 Stool
50 Stool
50 Stool
21 Stool
21 Stool
21 Stool
21 Stool
21 Stool
6608 Blood
6608 Stool
6608 Blood
6608 Blood
50 Stool
50 Stool
50 Stool
50 Stool
19 Blood
1359 Stool
1359 Stool
1359 Stool
1359 Stool
1359 Stool
1359 Stool
1359 Stool
1359 Stool
1359 Stool
1359 Stool
1359 Stool
1359 Stool
1359 Poul.
1359 Poul.
1359 Stool
1359 Stool
1359 Stool
1359 Stool
1359 Stool
1359 Stool
1359 Stool
353 Blood
6713 Stool
5 Blood
400 Blood
400 Stool
400 Blood
400 Stool
400 Stool
2337 Blood
2337 Blood
2122 Blood
5285 Stool
607 Blood
2927 Stool
354 Stool
354 Stool
354 Blood
1474 Stool
460 Blood
460 Stool
460 Stool
460 Stool
460 Blood
1923 Blood
257 Stool
257 Poul.
257 Poul.
257 Poul.
257 Poul.
257 Stool
257 Stool
257 Stool
257 Stool
257 Stool
257 Stool
257 Stool
257 Stool
257 Stool
257 Stool
257 Stool
257 Stool
257 Stool
257 Stool
257 Stool
257 Stool
257 Stool
257 Stool
257 Poul.
257 Poul.
257 Stool
257 Stool
257 Stool
257 Stool
257 Stool
257 Stool
257 Stool
257 Stool

LEGEND TO SUPPLEMENTARY FIGURE 1: Analysis of virulence factors in a national sample of Israeli *C. jejuni* isolates.

Type VI secretion system genes were detected in 7 clonal complexes (CCs). The virulence profiles of these CCs are presented. The dedrogram is based on wgMLST. Allelic distances are shown with a scaling factor of 10.

Clonal complex is denoted by color. Functional group, virulence factor, sequence type and source are denoted.
